# Neonicotinoids disrupt memory, circadian behaviour and sleep

**DOI:** 10.1038/s41598-021-81548-2

**Published:** 2021-01-21

**Authors:** Kiah Tasman, Sergio Hidalgo, Bangfu Zhu, Sean A. Rands, James J. L. Hodge

**Affiliations:** 1 School of Physiology, Pharmacology and Neuroscience, University of Bristol, Biomedical Sciences Building, University Walk, Bristol, BS8 1TD UK; 2 School of Biological Sciences, University of Bristol, Life Sciences Building, Tyndall Avenue, Bristol, BS8 1TQ UK

**Keywords:** Circadian rhythms and sleep, Ion channels in the nervous system, Learning and memory, Neural circuits, Behavioural ecology, Ecology, Genetics, Neuroscience, Physiology

## Abstract

Globally, neonicotinoids are the most used insecticides, despite their well-documented sub-lethal effects on beneficial insects. Neonicotinoids are nicotinic acetylcholine receptor agonists. Memory, circadian rhythmicity and sleep are essential for efficient foraging and pollination and require nicotinic acetylcholine receptor signalling. The effect of field-relevant concentrations of the European Union-banned neonicotinoids: imidacloprid, clothianidin, thiamethoxam and thiacloprid were tested on *Drosophila* memory, circadian rhythms and sleep. Field-relevant concentrations of imidacloprid, clothianidin and thiamethoxam disrupted learning, behavioural rhythmicity and sleep whilst thiacloprid exposure only affected sleep. Exposure to imidacloprid and clothianidin prevented the day/night remodelling and accumulation of pigment dispersing factor (PDF) neuropeptide in the dorsal terminals of clock neurons. Knockdown of the neonicotinoid susceptible Dα1 and Dβ2 nicotinic acetylcholine receptor subunits in the mushroom bodies or clock neurons recapitulated the neonicotinoid like deficits in memory or sleep/circadian behaviour respectively. Disruption of learning, circadian rhythmicity and sleep are likely to have far-reaching detrimental effects on beneficial insects in the field.

## Introduction

An estimated 84% of European crops are dependent on pollinators whose services are valued at > €22 bn/year and are essential to food security^1,2^. Globally, 35% of crop production relies on pollination and these services are valued at €153 bn/year^3,4^. However, populations of pollinating insects are declining dramatically. For instance flying insects have decreased by over 75% in Germany over the last 27 years^5^. Diminishing pollinator numbers are a serious threat to our food security^2,6^, with intensive use of insecticides being implicated in these losses^2,6^. However, a third of the global crop is lost to pests and without pesticides this loss could be 75%, keeping the demand for insecticides high^7,8^. The most common insecticides worldwide are neonicotinoids, which account for 24% of the global insecticide market valued at $1 billion/year^8,9^. Neonicotinoids are highly efficacious insecticides however they lack specificity, affecting both target pest species such as aphids and non-target beneficial insects, such as bees. They share a mechanism of action, being agonists of nicotinic acetylcholine receptors (nAChR), the main neurotransmitter in the insect nervous system. They also display target site cross-resistance in pests, diminishing their effectiveness as insecticides and unfortunately encouraging application of increasing concentrations^9,10^. They were branded safe compared to previous insecticides and because they have little effect on mammalian nAChRs^9,10^. However, few precursive safety tests were performed/published on beneficial insects, for which neonicotinoids are now known to be potent neurotoxins with well-documented lethal and sub-lethal effects^1,7,9,11^. Therefore, continued intensive use is likely to have severe consequences on insect species numbers, with knock-on as well as direct effects on the ecosystem, aquatic life, birds and mammals including potential toxicity to humans^1,12–14^. This has resulted in the European Union (EU) banning the nitrome neonicotinoids; imidacloprid, clothianidin and thiamethoxam (a prodrug for clothiandin^15^) in 2017 followed by the cyanoimine neonicotinoid, thiacloprid in 2020^16^. Despite this the neonicotinoids remain the most widely used class of insecticide globally and a number of studies show there has been no decrease in the quantity of banned neonicotinoids found in different populations of honey and bumble bee across Europe a year after the ban^17,18^. Furthermore, some national governments have granted multiple exemptions for the spraying of oil seed rape and a number of other applications^19^. Furthermore neonicotinoids have high solubility and persistence in the environment^1^. Therefore, despite the current EU ban, insects are still at risk of neonicotinoid exposure.

In the field, concentrations of neonicotinoids encountered by non-target insects are typically between 1 and 51 μg/L for seed treated crops and 61–127 μg/L for sprayed crops^11^. Concentrations as low as 1 µg/L (or 1 part per billion (ppb)) can cause significant behavioural effects due to the high potency of neonicotinoids, these include reduced foraging motivation^20^ and circadian rhythms^21^ in the bumblebee *Bombus terrestris*^20^. The potential sub-lethal effects of neonicotinoids are very far-reaching because of the central role of nAChR in synaptic neurotransmission in the insect brain^9,10^. Neonicotinoids cause this ligand-gated ion channel to open, thereby depolarising the neuron and increasing excitability. Prolonged exposure to the depolarising agonist may result in depolarising block, where the protracted depolarisation causes inactivation of the voltage sensitive Na^+^ channels required for action potential firing and the long-term agonist exposure causes nAChR desensitization^10,22^. These effects are likely to have pronounced effects on memory formation and consolidation required for effective foraging in many pollinating insects.

Previous research in *Drosophila* demonstrated that both the Kenyon cells (which constitute the insect memory centre called the mushroom body^23^) and their output neurons (which mediate memory valence) are nicotinic^24^. The mushroom body is also involved in sleep in insects^25^. In honeybees, sub-lethal neonicotinoids electrically inactivated^22^ and decreased the synaptic density^26^ of mushroom body neurons and resulted in disrupted olfactory memory^22^. In bumblebees, exposure during development reduced mushroom body growth, causing learning deficits in adults^27^. Neonicotinoids also reduced honeybee antennal lobe Ca^2+^ responses and caused sensory deficits^28^, potentially contributing to olfactory memory deficits.

Memory formation is also reliant on circadian rhythms^29,30^ and sleep^31,32^. Work in honeybees and bumblebees has shown that neonicotinioids can reduce behavioural rhythmicity and disrupt sleep behaviour in these important pollinators^21,33^. The mode of action of neonicotinoids on circadian rhythmicity and sleep is unknown. However, work in *Drosophila* has shown nAChR signalling is required for setting of the central clock and communication between the light sensing organs and the central clock^34–37^. The timing of sleep/wake cycles is also determined by the circadian clock^38^ with the key clock neurons that mediate arousal expressing nAChRs^35,39^.

The pacemaker neurons of the insect clock consist of the pigment dispersing factor (PDF) neuropeptide expressing small and large ventral lateral neurons (s- and l-LNvs). The s-LNvs maintain rhythmicity in constant conditions and set the pace of the insect clock via PDF signalling^40^. The LNvs express both nicotinic and muscarinic acetylcholine receptors^39,41,42^. They receive ACh from the visual circuit including the lamina, with the s-LNvs also receiving ACh-mediated light input information from the Hofbauer-Buchner (HB) eyelets^34,35^. These excitatory signals regulate the electrical excitability of the LNvs which is required for circadian function^43^, with the neurons being more depolarised and having increased firing rate in the day than at night^44^. These day/night differences in excitability help sustain the molecular oscillation of clock genes in constant conditions as well as regulating day/night changes in s-LNv terminal remodelling and PDF release necessary for robust behavioural rhythmicity^43^. The s-LNv dorsal terminals exhibit circadian remodelling, with their terminals being more branched during the day than at night^45^ and having higher PDF accumulation in the day than at night^43,46^. LNv expression of a GCaMP Ca^2+^ reporter showed that 4 µg/l clothiandin caused a transient (~ 1 min) increase in Ca^2+^ dependent fluorescence^33^. While similar experiments using a nAChR promoter Gal4 line (which drives expression wherever the endogenous nAChR is expressed) to express GCaMP showed that 2.5 µg/L imidacloprid blocked nAChR agonist (e.g. carbachol) evoked Ca^2+^ responses in larval neurons. A 2 h exposure of 2.5 µg/L imidacloprid increased gut and brain levels of superoxide reactive oxygen species and decreased mitochondrial function and ATP levels^47^. Chronic exposure of 4 µg/L imidacloprid led to degeneration of the fly retinae and loss of visual acuity, providing further evidence that field relevant sublethal concentrations of neonicotinoids bring about irreversible effects at the neuronal level.

The circuitry and molecular components of the mushroom body and the clock identified in *Drosophila* are shown to be highly conserved amongst insects^25,48,49^ making it a powerful model to test the effects of neonicotinoids on memory, circadian behaviour and sleep. *Drosophila* have ten different nAChR subunits most of which are highly conserved across insect species, making it probable that a neurotoxin selected for its high potency to target insect nAChRs will affect the equivalent nAChR in beneficial insects^50^. Whilst the subunit conformation and location of neonicotinoid susceptible nAChRs is still largely unknown, in *Drosophila* the subunits Dα1 and Dβ2 have been shown to play a role in neonicotinoid susceptibility and resistance, with mutations in these conveying both neonicotinoid resistance and significant fitness costs^9,10,51^. Given the power of *Drosophila* as a model system, and the likely generalisation provided by conservation of nAChR function across insects, we tested the sub-lethal effect of field-relevant concentrations of four major neonicotinoids on *Drosophila* memory, circadian rhythms and sleep.

## Results

### Neonicotinoids reduce longevity, offspring viability and climbing ability in *Drosophila*

Field relevant concentrations of neonicotinoids cause a range of lethal and sub-lethal effects in bees^1,52^. In order to validate the use of *Drosophila* as a model for these lethal and sub-lethal effects*,* we fed field relevant concentrations of four commonly used neonicotinoids to *Drosophila* and determined their effect on longevity, offspring viability and climbing ability. As in pollinators, longevity, fecundity and mobility were all affected by neonicotinoid exposure in *Drosophila*^1,53–55^. The mean lifespan of control flies was 49 days while exposure to a field relevant concentration of 10 µg/L clothianidin causing a reduction to 28 days, imidacloprid and thiamethoxam to 36 days, and thiacloprid, to 39 days (Supplementary Fig. S1). The number of eggs laid were increased by 1 or 10 µg/L of thiamethoxam or clothianidin or 100 µg/L imidacloprid (Supplementary Fig. S2). However, the overall viability of the eggs was reduced, with 100 µg/L clothianidin, thiamethoxam or thiacloprid and 10 or 100 µg/L imidacloprid reducing the actual proportion of eggs that subsequently completed development and successfully eclosed as adults (Supplementary Fig. S3). Likewise, field relevant concentrations of imidacloprid, clothianidin and thiamethoxam (10 and 50 µg/L) all reduced locomotor performance, tested via a negative geotaxis climbing assay, whilst thiacloprid had no effect on locomotion at these concentrations (Supplementary Fig. S4).

### Neonicotinoids reduced memory in *Drosophila*

**Figure 1:**
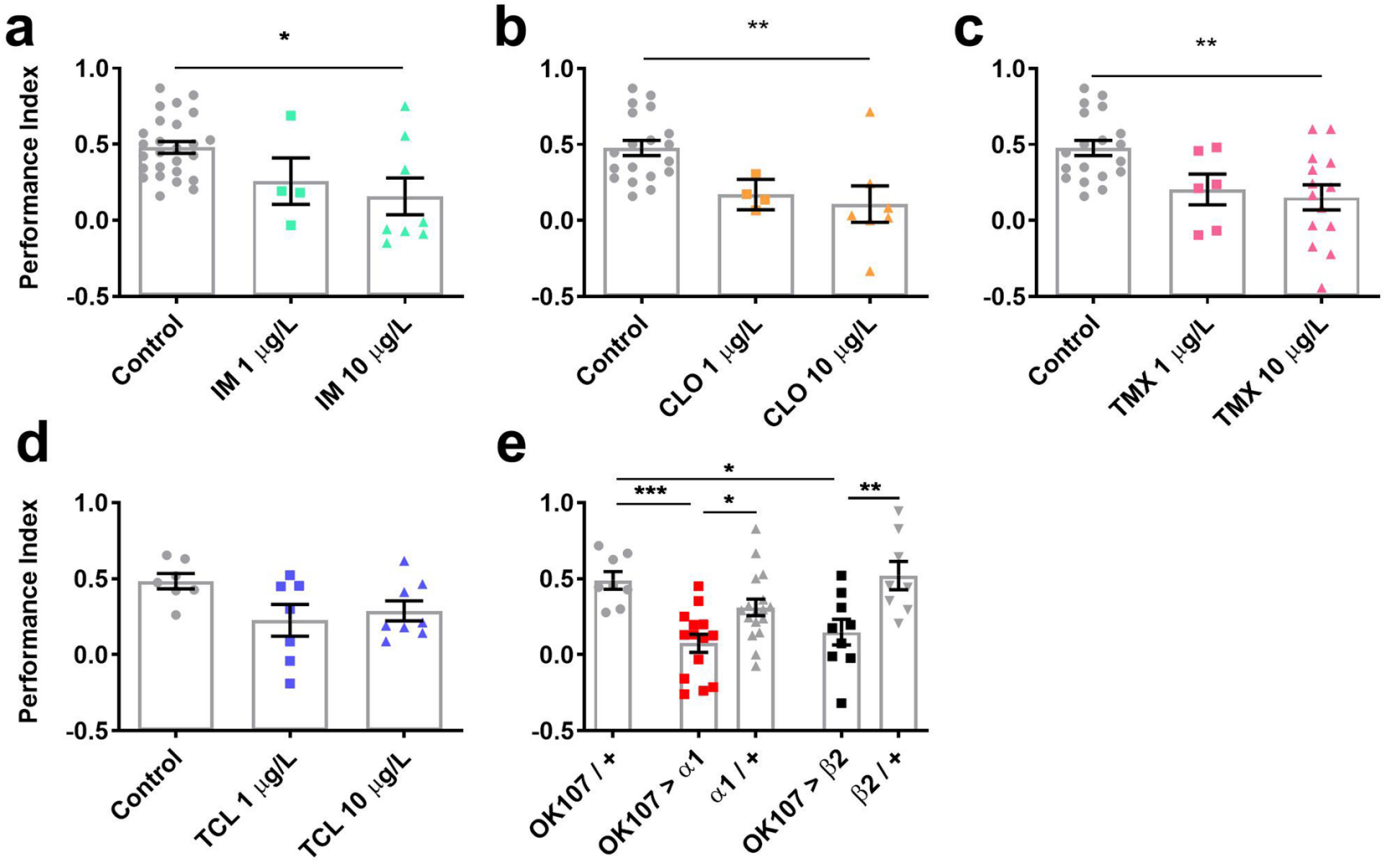
Field relevant concentrations of neonicotinoids or knockdown of Dα1 or Dβ2 in the mushroom bodies reduced 1 h memory. 1 h memory was reduced in flies exposed to field relevant concentrations of 1 or 10 μg/L (**a**) imidacloprid (IM) (*χ*^*2*^_2_) = 7.3, *p* = 0.026), (**b**) clothianidin (CLO) (*χ*^*2*^_2_ = 12.4, *p* = 0.002), (**c**) thiamethoxam (TMX) (*χ*^*2*^_2_ = 9.6, *p* = 0.008) and not in (**d**) thiacloprid (TCL) (*χ*^*2*^_2_ = 5.0, *p* = 0.084). (**e**) Likewise, 1 h memory was reduced in flies with *RNAi* mediated knockdown of Dα1 (*OK107-Gal4* > *uas-nAChR-Dα1*) or Dβ2 (*OK107-Gal4* > *uas-nAChR-Dβ2*) throughout the mushroom body (*F*_2,20_ = 4.6, *p* = 0.023) and compared to genotype controls. Each data point represents ~ 100 flies, n ≥ 4 per treatment. Graphs show mean ± standard error of the mean (SEM) (post hoc pairwise comparisons: *p* ≤ 0.05*, *p* ≤ 0.01**, *p* ≤ 0.001***, *p* ≤ 0.0001****). The same tests, error bars and *p* values were used throughout.

Olfactory associative memory is critical for foraging pollinators and has been shown to be disrupted by neonicotinoids in bees^56^. In order to see if field relevant concentrations of imidacloprid, clothianidin, thiamethoxam and thiacloprid had a similar effect on flies, 1 h memory (Fig. 1) was assessed using *Drosophila* olfactory shock conditioning^57^. Imidacloprid, clothianidin and thiamethoxam all reduced memory at 10 µg/L (Fig. 1a–c) whilst thiacloprid left memory intact (Fig. 1d). Sensory controls showed that none of the neonicotinoids tested reduced the ability of flies to sense either the odours or the aversive stimuli (Supplementary Fig. S5). In order to localise the effect of nAChR mis-regulation on memory, we expressed *RNAi* transgenes to the neonicotinoid sensitive nAChR Dα1 and Dβ2 subunits, which had previously been shown to block nAChR mediated Ca^2+^ responses in the mushroom body memory circuit^24^. In order to further validate the *RNAi* lines we performed qRT-PCR on brains extracted from flies that pan-neuronally expressed (with *elav-Gal4*) the Dα1 or Dβ2 transgenes and measured their *Dα1* or *Dβ2* mRNA expression which was shown to be significantly decreased by 33% and 58%, respectively (Supplementary Fig. S10). Knockdown of Dα1 and Dβ2 throughout the mushroom body significantly reduced memory to a similar level to that caused by neonicotinoid exposure (Fig. 1e), confirming the importance of these subunits in mushroom body mediated memory and the effect of neonicotinoids on this behaviour.

### Neonicotinoids decrease circadian rhythmicity and sleep in *Drosophila*

**Figure 2:**
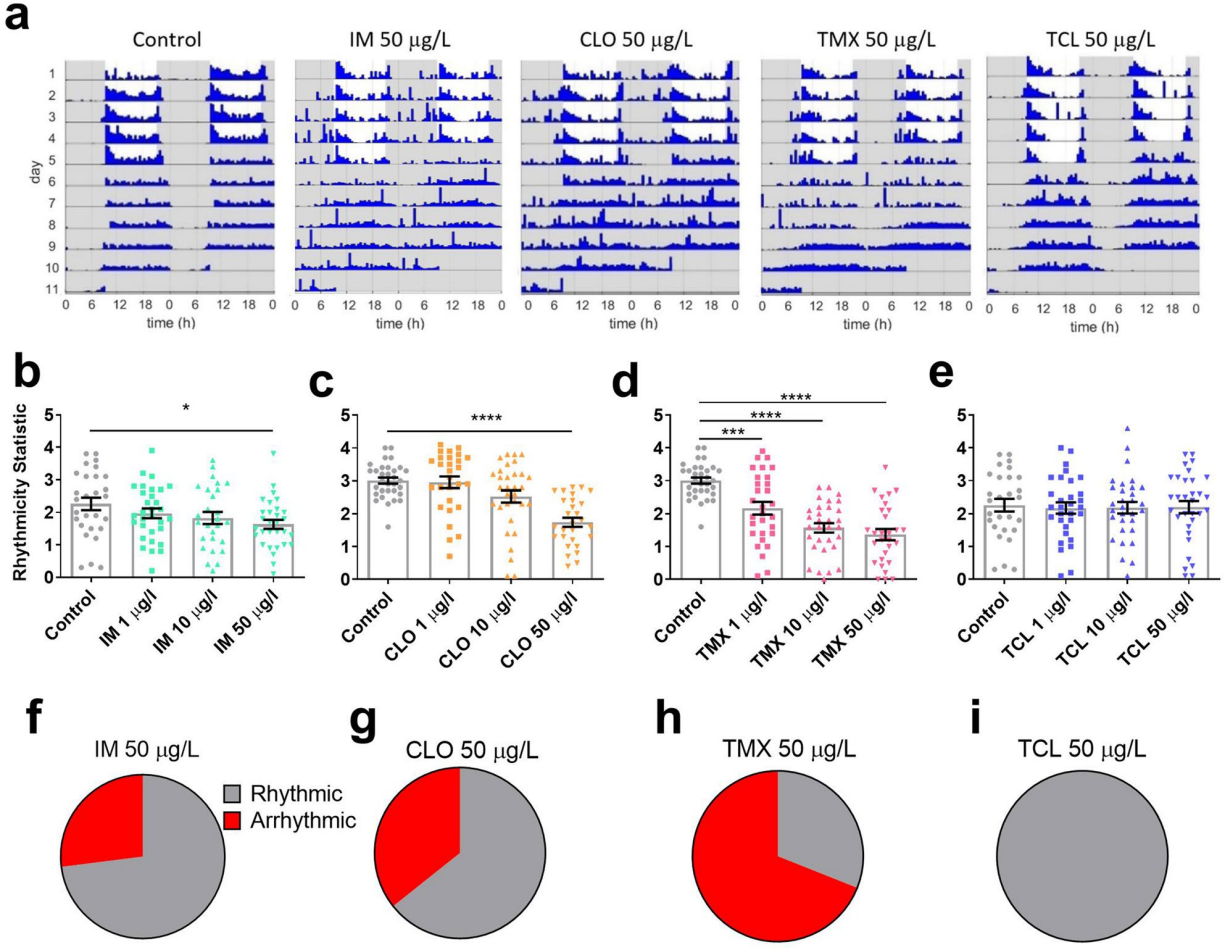
Field relevant concentrations of neonicotinoids reduced behavioural rhythmicity. (**a**) Representative actograms of the activity of single flies for control or 50 μg/L imidacloprid, clothianidin, thiamethoxam or thiacloprid. Blue bars indicate activity, grey background indicates lights off, white background lights on. Mean rhythmicity for flies exposed to 1, 10 or 50 μg/L (**b**) IM (*F*_3,112_ = 2.5, *p* = 0.060), (**c**) CLO (*F*_3,116_ = 14.2, *p* < 0.001), (**d**) TMX (*F*_3,118_ = 23.7, *p* < 0.001) and (**e**) TCL (*F*_3,118_ = 0.05, *p* = 0.987). Each data point represents a single fly, n = 28–32 flies per treatment. Pie charts show the increase in the proportion of the population who were arrhythmic (rhythmicity statistic (RS) ≤ 1.5) for 50 μg/L: (**f**) IM, (**g**) CLO, (**h**) TMX and (**i**) TCL, compared to controls.

The effect of neonicotinoids on circadian rhythmicity and sleep was then measured as these behaviours are known to be regulated by nAChR signalling^25,35,36,39^. The effect of field relevant concentrations of neonicotinoids on circadian rhythms was tested using the *Drosophila* Activity Monitor (DAM2, Trikinetics Inc, USA)^58^. Imidacloprid, clothianidin and thiamethoxam all reduced circadian rhythmicity (Fig. 2), with flies showing greatest sensitivity to thiamethoxam, which caused a reduction in mean rhythmicity at 1, 10 and 50 μg/L (Fig. 2d) while clothianidin and imidacloprid caused a reduction in mean rhythmicity at 50 μg/L (Fig. 2b,c). Any concentration of the three neonicotinoids caused an increase in the proportion of flies that were arrhythmic (rhythmicity statistic (R.S.) ≤ 1.5) compared to controls (Fig. 2f–i, Supplementary Table S1). Again, thiacloprid appeared not to have sub-lethal effects, with field-relevant concentrations leaving circadian rhythmicity intact (Fig. 2e,i). None of the neonicotinoids affected the free-running period length (Supplementary Fig. S6). Under LD conditions, the effects on rhythmicity were much smaller, with RS remaining high and only IM and TMX having an effect (Supplementary Fig. S7).

**Figure 3:**
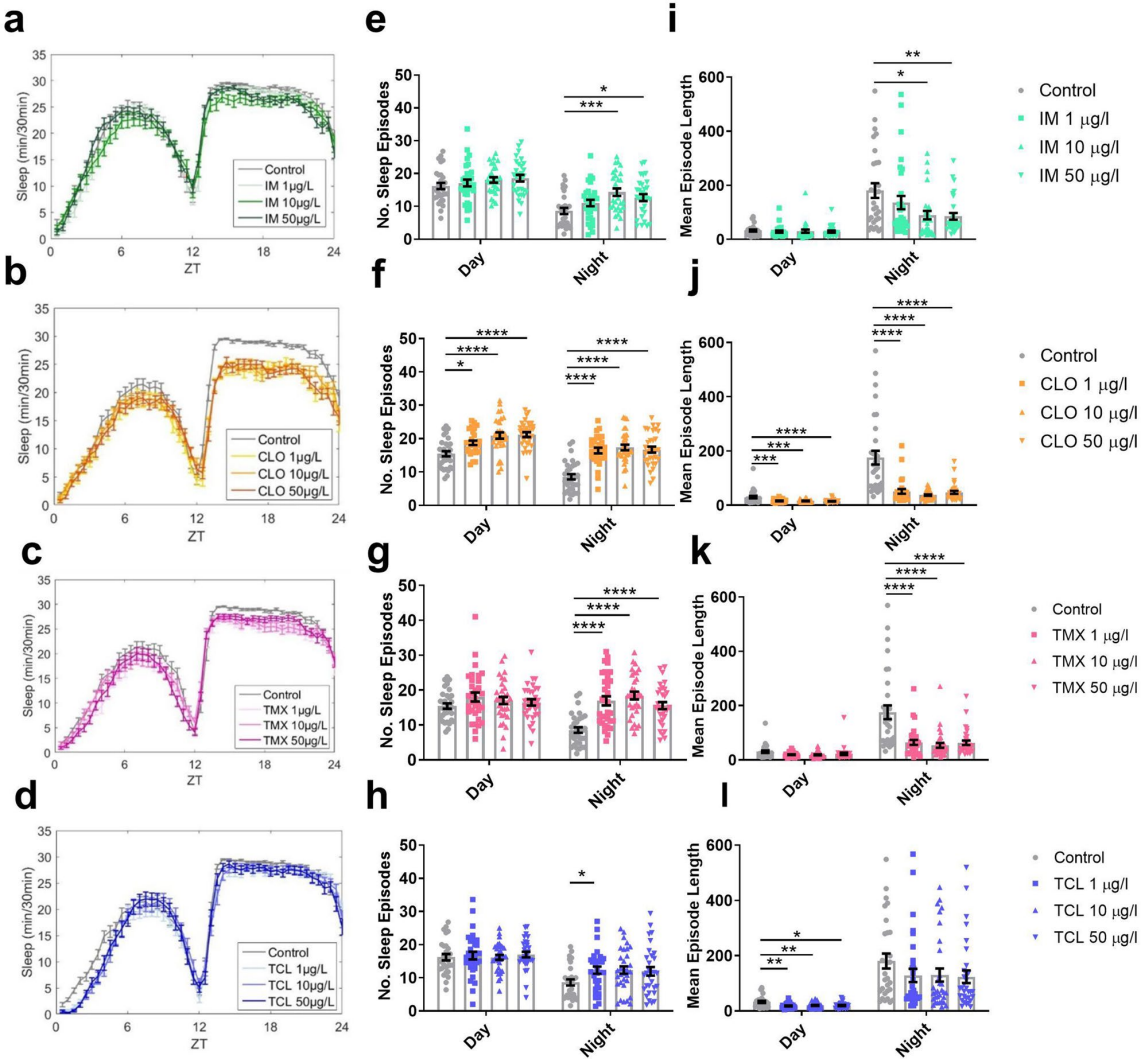
Field relevant concentrations of neonicotinoids disrupted sleep behaviour. Sleep plots showing the total sleep achieved per 30 min bin over the 24 h period (zeitgeber time (ZT)) for flies exposed to 1, 10 or 50 µg/L of (**a**) imidacloprid, (**b**) clothianidin, (**c**) thiamethoxam or (**d**) thiacloprid. The number of (no.) of sleep episodes initiated in (**e**) IM, day (*F*_3,114_ = 1.2, *p* = 0.320) and night (*F*_3,114_ = 5.5, *p* = 0.001), (**f**) CLO, day (*F*_3,120_ = 11.5, *p* < 0.001) and night (*F*_3,120_ = 25.0, *p* < 0.001), (**g**) TMX, day (*F*_3,124_ = 1.1, *p* = 0.344) and night (*F*_3,124_ = 17.0, *p* < 0.001) or (**h**) TCL, day (*F*_3,120_ = 0.2, *p* = 0.872) and night (*F*_3,120_ = 3.0, *p* = 0.034). Mean length (in minutes) of sleep episodes initiated in (**i**) IM, day (*F*_3,114_ = 0.2, *p* = 0.889) and night (*F*_3,114_ = 4.5, *p* = 0.005), (**j**) CLO, day (*F*_3,120_ = 9.9, *p* < 0.001) and night (*F*_3,120_ = 21.8, *p* < 0.001), (**k**) TMX, day (*F*_3,124_ = 2.5, *p* = 0.061) and night (*F*_3,124_ = 15.7, *p* < 0.001) or (**l**) TCL, day (*F*_3,120_ = 5.2, *p* = 0.002) and night (*F*_3,120_ = 2.0, *p* = 0.121). Each data point represents a single fly, *n* = 28–32 flies per treatment. CLO and TMX were run together with one set of controls, whilst IM and TCL were run together with another control group.

Sleep was also monitored using the DAM system, with bouts of inactivity lasting more than 5 min qualifying as sleep^37^. Field relevant concentrations of all four neonicotinoids caused fragmentation of sleep, arising from sleep formed of a greater number of sleep episodes (Fig. 3e–h) of shorter length compared to control (Fig. 3i–l). This effect was greatest for clothianidin, where 1, 10 and 50 μg/L caused fragmentation of both daytime and night-time sleep (Fig. 3f,j) resulting in a reduction of night-time sleep (Fig. 3b, Supplementary Fig. S8). Thiamethoxam and imidacloprid had a similar effect (Fig. 3e,g,i,k) but only for night-time sleep (Fig. 3a,c, Supplementary Fig. S8). Thiacloprid caused an increase in the number of night-time sleep episodes (Fig. 3h) and unlike the other neonicotinoids, caused a loss in daytime sleep (Fig. 3d, Supplementary Fig. 7) at every concentration tested, due to a reduction in daytime sleep episode length (Fig. 3l). This is likely due to the increase in daytime sleep latency observed in thiacloprid treated flies (Supplementary Fig. S9).

**Figure 4:**
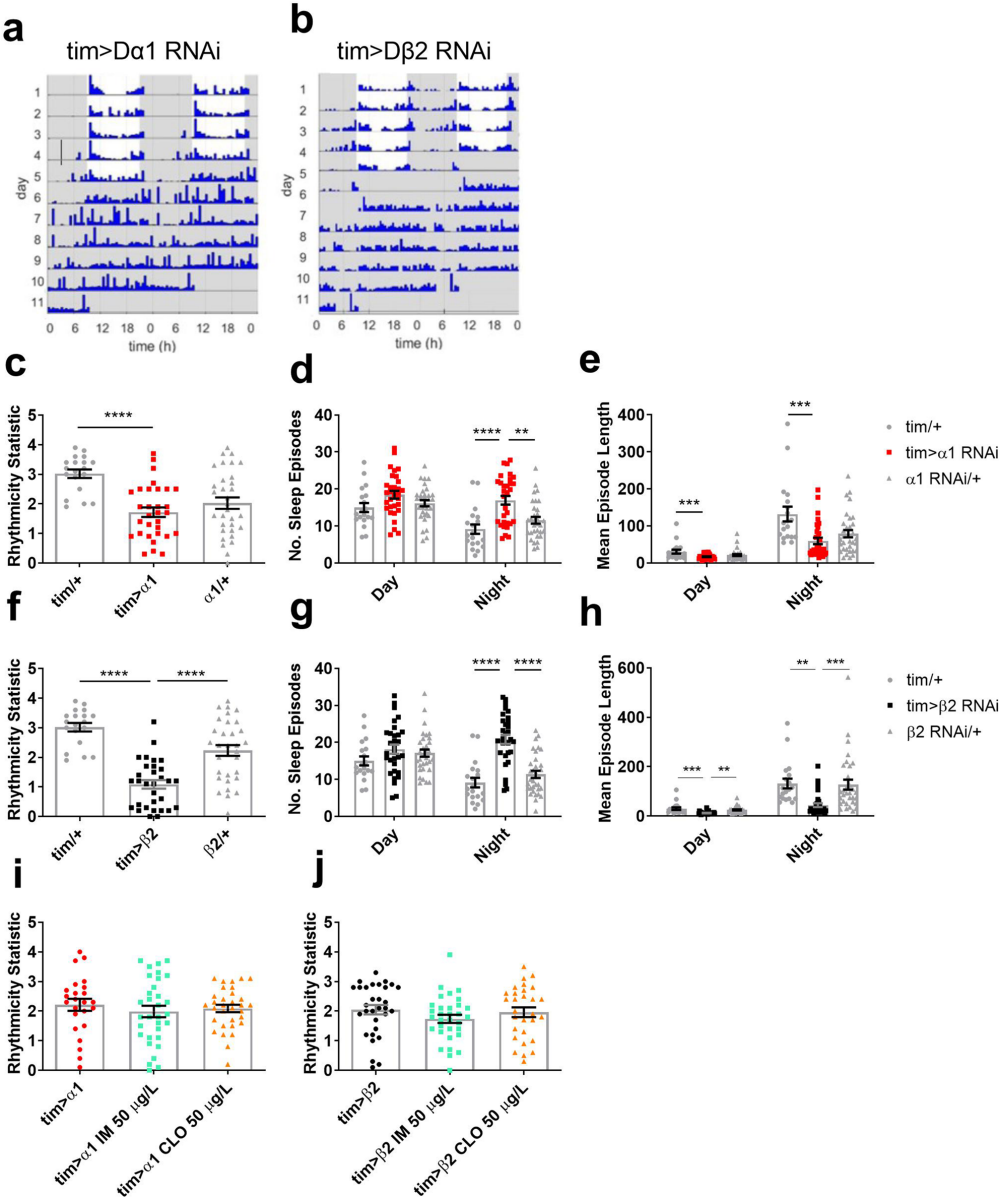
Knockdown of Dα1 or Dβ2 in the clock bearing cells disrupts circadian rhythmicity and sleep with no further effect by addition of neonicotinoids. Representative actograms for (**a**) Dα1 knock down (*tim-Gal4* > *uas-nAChR-Dα1*) and (**b**) Dβ2 knockdown (*tim-Gal4* > *uas-nAChR-Dβ2.* Effects of knocking down *Dα1* in clock bearing cells on (**c**) rhythmicity (RS) (*F*_2,79_ = 11.8, *p* < 0.001), (**d**) number (no.) of sleep episodes in day (*F*_2,79_ = 2.9, *p* = 0.063) and night (*F*_2,79_ = 12.3, *p* < 0.001) and (**e**) mean episode length in day (*F*_2,79_ = 5.1, *p* = 0.008) and night (*F*_2,79_ = 8.3, *p* = 0.001). Effects of knocking down *Dβ2* in clock bearing cells on (**f**) rhythmicity (*F*_2,79_ = 31.5, *p* < 0.001), (**g**) no. of sleep episodes in day (*F*_2,79_ = 1.6, *p* = 0.211) and night (*F*_2,79_ = 28.2, *p* < 0.001) and (**h**) mean episode length in day (*F*_2,79_ = 11.2, *p* < 0.001) and night (*F*_2,79_ = 9.4, *p* < 0.001). Each data point represents a single fly, *n* = 19–32 flies per treatment. There was no additive effect of 50 µg/L of IM and CLO on (**i**) *tim* > *α1* (*F*_2,85_ = 0.4, *p* = 0.677) and (**j**) *tim* > *β2* (*F*_2,89_ = 1.1, *p* = 0.336). Each data point represents a single fly, n = 24–32 flies per treatment.

In order to localise the effects of neonicotinoids on sleep and circadian behaviour we knocked down Dα1 or Dβ2 in *tim*-expressing cells throughout the neuronal clock network (Fig. 4). This resulted in reduced behavioural rhythmicity (Fig. 4b,f) and shorter night-time sleep episodes (Fig. 4g,h) in *Dβ2* knockdown flies, and increased the number of sleep episodes seen in both *Dα1* and *Dβ2* knockdown flies (Fig. 4d,g). This again showed that loss of these subunits caused similar behavioural disruption as neonicotinoid exposure, suggesting a functional nAChR containing these subunits mediates the in vivo effects of these insecticides. In order to test this was indeed the case, *RNAi* flies were exposed to 50 μg/L of imidacloprid or clothianidin, a concentration sufficient to reduce rhythmicity in control flies. On flies that already had their Dα1 or Dβ2 blocked genetically by expression of subunit specific *RNAi* expression throughout the *tim*-expressing clock cells, we found this caused no further loss of rhythmicity (Fig. 4i,j), confirming that neonicotinoid’s in vivo effects were mediated through a receptor containing one or both of subunits in the clock.

### Neonicotinoids remove day/night differences in synaptic remodelling and PDF accumulation in the s-LNvs dorsal terminals

**Figure 5:**
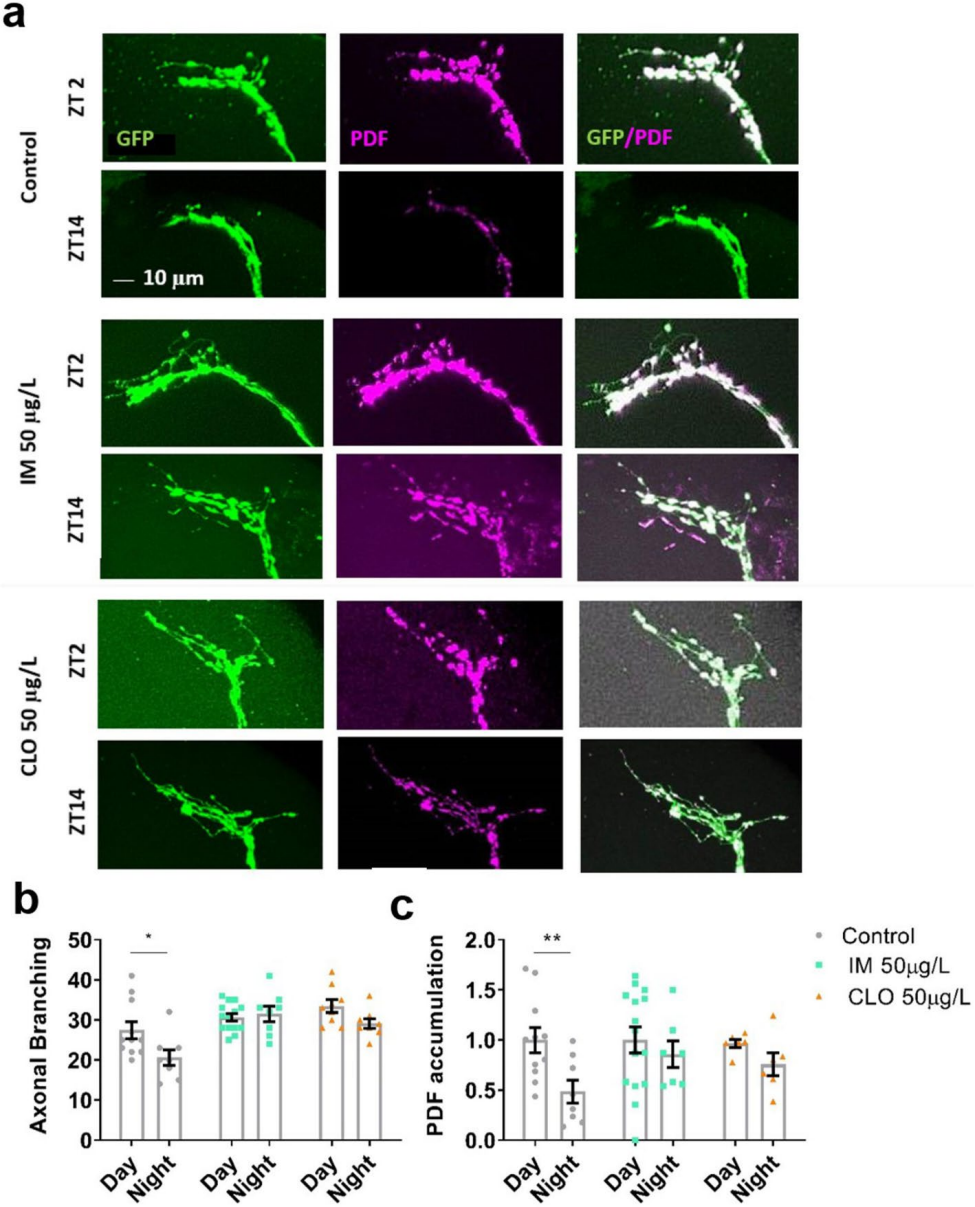
Field relevant concentrations of neonicotinoids disrupt the day/night remodelling and PDF cycling in the s-LNv clock neuron dorsal terminals. **(a**) Representative confocal images of the s-LNv dorsal terminals for control and treated (50 µg/L IM or CLO) flies in the day (ZT2 i.e. 11am) and night (ZT14 i.e. 11 pm). (**b**) s-LNv dorsal terminal branching complexity is greater in the day than at night for control flies (*t*_17_ = 2.3, *p* = 0.036). The day/night differences in complexity is removed in flies exposed to 50 µg/L of IM (*t*_14_ = 2.1, *p* = 0.055) or CLO (*t*_15_ = 2.1, *p* = 0.052). (**c**) Accumulation of PDF in dorsal terminals is greater in the day than at night in control flies (*t*_17_ = 2.9, *p* = 0.010), treatment with 50 µg/L IM (*t*_13_ = 1.0, *p* = 0.332) or CLO (*t*_14_ = 2.1, *p* = 0.054) removed this day/night difference in PDF levels. Each data point represents a single brain, n = 6–15 brains.

**Figure 6:**
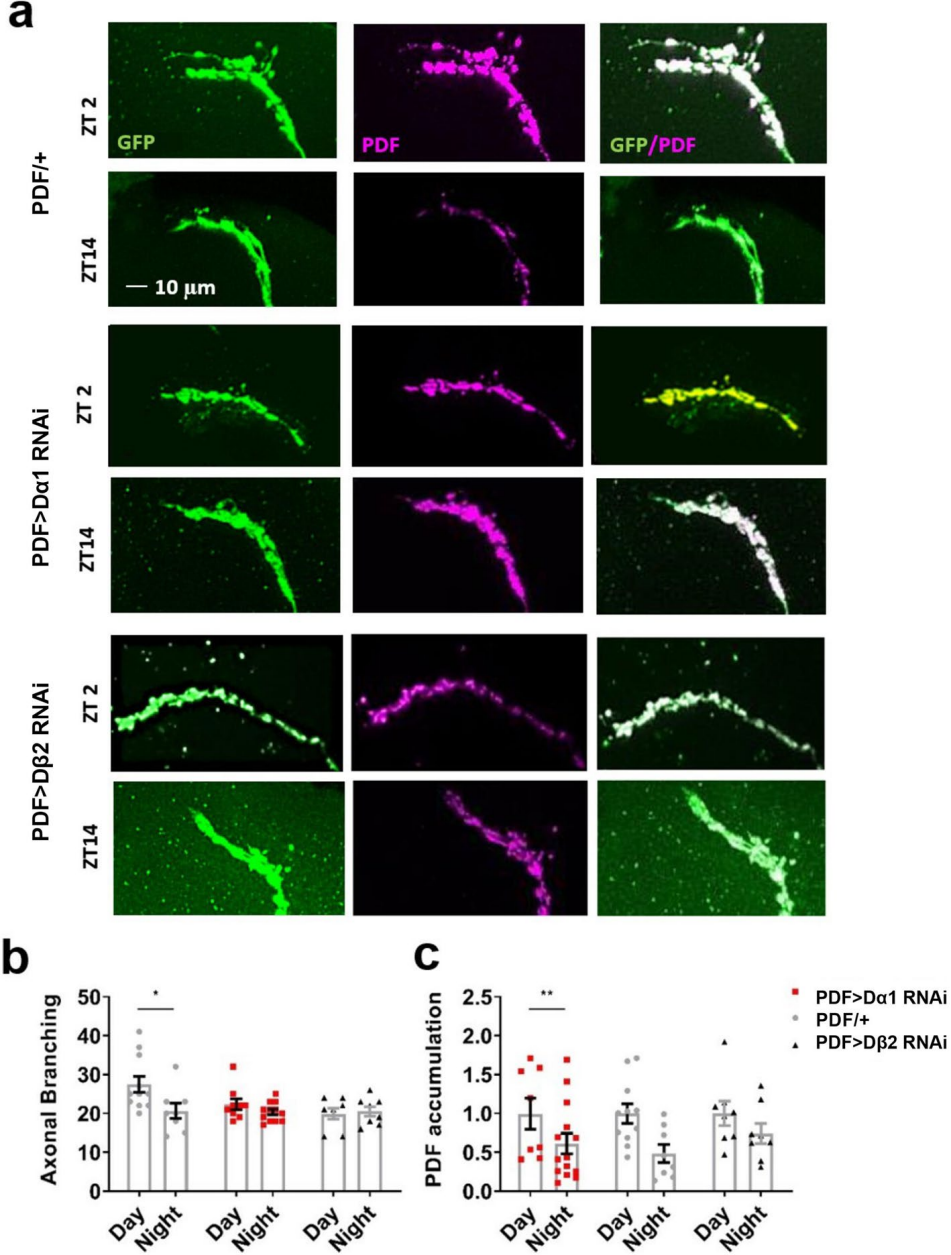
Knockdown of Dα1 or Dβ2 disrupted the day/night remodelling and PDF cycling of the s-LNv dorsal terminals. (**a**) Representative confocal images of the s-LNv dorsal terminals of control flies (*PDF-Gal4/* +) and flies with Dα1 (*PDF-Gal4* > *uas-nAChR-Dα1-RNAi*) and Dβ2 (*PDF-Gal4* > *uas-nAChR-Dβ2-RNAi*) knocked down in LNv clock neurons taken in the day (ZT2) and night (ZT14). (**b**) The s-LNv dorsal terminals of control flies showed greater branching complexity in the day than at night (*t*_17_ = 2.3, *p* = 0.036), this day/night difference in terminal complexity was removed in *PDF* > *Dα1-RNAi* (*t*_19_ = 1.4, *p* = 0.183) and *PDF* > *Dβ2-RNAi* (*t*_13_ = -0.7, *p* = 0.515) flies. (**c**) PDF accumulation in the s-LNv dorsal terminals was greater in the day than at night for control flies (*t*_17_ = 2.9, *p* = 0.010), but not in *PDF* > *Dα1-RNAi* flies (*t*_19_ = 1.8, *p* = 0.089) and *PDF* > *Dβ2-RNAi* flies (*t*_14_ = 1.3, *p* = 0.218). Each data point represents a single brain, n = 6–15 brains.

To further characterise the mechanism by which neonicotinoids disrupt circadian rhythms day/night differences in synaptic remodelling and PDF cycling of the sLNv dorsal terminals were investigated. As previously reported^45^, in control flies the terminals were more branched and had higher accumulation of PDF in the day than at night (Fig. 5a–c). In contrast, flies exposed to 50 μg/L imidacloprid or clothianidin showed no difference between day and night synaptic terminal branching or PDF accumulation (Fig. 5a–c). Likewise, in flies with knockdown of either Dα1 or Dβ2 nAChRs in the PDF neurons, branching and PDF accumulation again showed no difference between day and night (Fig. 6a–c). Therefore the pharmacological and genetic manipulation of LNv nAChRs both similarly block the circadian output of these PDF expressing pacemaker neurons resulting in compromised circadian behaviour observed.

## Discussion

We showed that field relevant concentrations of all neonicotinoids tested had significant lethal effects on *Drosophila,* including decreased viability and shortened lifespan. In contrast the behavioural or sub-lethal effects on flies differed between the neonicotinoids with the nitro group-containing imidacloprid, clothianidin and thiamethoxam disrupting memory, locomotion, sleep and circadian behaviour, in contrast the cyano-group containing thiacloprid only caused fragmentation and reduction in sleep leaving the other behaviours intact. Therefore, thiacloprid appears to be less disruptive to behaviour than the other neonicotinoids tested. However its effects on sleep revealed significant sub-lethal effects even at the lowest level (1 ppb) reported to have sub-lethal effects by any neonicotinoid. In addition it decreased viability and caused early death, providing further evidence in support of the EU’s extension of the neonicotinoid ban to thiacloprid. Clothianidin and thiamethoxam showed the greatest effects, which is consistent with them being full agonists at nAChRs and many other studies in pollinators finding them to be more toxic and potent than the partial agonist imidacloprid^9,52,59^. The sub-lethal effects we report in *Drosophila* are also consistent with those reported for pollinators that show the nitro-containing neonicotinoids (clothianidin, thiamethoxam and imidacloprid) are generally more toxic than the cyano-group containing neonicotinoids (thiacloprid)^1,9–11,52^.

The effects of field-relevant concentrations of neonicotinoids on memory in *Drosophila*, reiterates the conserved toxicity and sub-lethal effects of the insecticides on non-target insects. That lack of effect of neonicotinoids on fly olfaction and to shock reactivity, confirmed that the neonicotinoids interfered with memory formation itself as opposed to detection of cues. This was also supported by the data showing that knockdown of the neonicotinoid susceptible Dα1 or Dβ2 subunits in just the mushroom body was sufficient to cause similar memory deficits as observed in the neonicotinoid exposed flies. The Kenyon cells of the mushroom body are cholinergic^59^, contain nAChRs^60^ and the postsynaptic mushroom body output neuron also signal via nAChRs^24^. Therefore, neonicotinoids can act at multiple nAChR synapses in the mushroom body circuit, disrupting the plasticity-relevant signals for memory formation. Furthermore, in bees, field relevant concentrations of neonicotinoids disrupted mushroom body-mediated olfactory memory, electrically inactivated Kenyon cells^22^, decreased their synaptic density^26^ and reduced antennal lobe Ca^2+^ responses upstream of the mushroom body^27^. Additionally, neonicotinoids have been shown to interrupt mushroom body development^27^, providing a further route for memory disruption. Our results extend existing data from pollinators showing neonicotinoid disrupt memory by showing for the first time that the disruption is mediated by nAChRs containing Dα1 or Dβ2 subunits in the mushroom body. Furthermore, given that neonicotinoids appear to disrupt circadian rhythmicity, and given that there is circadian regulation of memory formation with time of day influencing performance, neonicotinoid-induced disruption to the clock may contribute to the memory deficits^61^. The time-of-day differences in mushroom body memory are thought to result from day/night differences in PDF and PDFR signalling^30^, therefore the loss sLNv dorsal terminal PDF rhythmic signalling of PDF caused by the neonicotinoids may provide another route to the insecticide induced reductions in memory we report.

Similarly, the sleep and circadian effects caused by neonicotinoid exposure appear to be due to the neonicotinoids acting directly upon the clock. Knockdown of Dβ2 nAChR subunit in the clock neurons resulted in a similar disruption of rhythmicity as exposure to field-relevant concentrations of neonicotinoids, whilst knockdown of Dα1 or Dβ2 caused changes to sleep behaviour reflecting those seen in neonicotinoid exposed flies. This suggests that Dα1 and Dβ2 mediate the effects of neonicotinoids on clock neurons resulting in the disruption of circadian rhythms and sleep. Consistent with this, exposure of Dβ2 knockdown flies to imidacloprid or clothianidin caused no further effect on circadian rhythmicity confirming that Dβ2 in clock neurons mediates the in vivo effect of neonicotinoids on circadian rhythms.

This is consistent with studies that have shown the LNvs usually receive excitatory ACh inputs from the light-sensing organs^34,35^ and express nAChRs^39,42^, further field relevant concentrations of neonicotinoids have been shown to directly increase LNv neural activity^23^. The electrical state of these pacemaker neurons influences their circadian output including the circadian remodelling of the s-LNv dorsal terminals and circadian abundance of PDF^43,62^, with the release of this neuropeptide being necessary for behavioural rhythmicity^40^. We found that neonicotinoid exposure caused a loss of PDF cycling and terminal morphological plasticity, with the terminals remaining in a branched, day state continuously, suggesting that the agonist effect of neonicotinoids disrupt normal day-night differences in the electrical state of these neurons that is required for circadian rhythms^35,36,43,44,58^. Indeed, nAChR synaptic signalling is involved in the rhythmic firing of action potentials in clock neurons of *Drosophila* and other insects^36,42,63^. Furthermore field relevant concentrations of imidacloprid have also shown to cause degeneration of retinae and reduced visual acuity, providing a further possible route for neonicotinoids to disrupt light input into the clock, and hence the structural and behavioural changes observed here^47^.

Knock-down of the neonicotinoid susceptible nAChR subunits Dα1 or Dβ2 also removed day-night differences in the terminals reminiscent of the changes seen in flies with electrically silenced LNvs^62^. The agonist action of neonicotinoids on nAChRs on the LNvs we and others have demonstrated^23^ also explains the sleep disruption we reported as the l-LNv electrical state is vital to their role as arousal neurons, with l-LNv hyperexcitation leading to sleep defects such as loss of night-time sleep and shorter sleep episodes^37,64^, effects recapitulated in the neonicotinoid exposed flies.

The high degree of structural and functional conservation of nAChRs between flies and bees^9,50,65^, and the conserved lethal and sub-lethal effects of reduced viability, longevity, locomotion, memory, circadian rhythmicity and sleep we show in flies and as reported in bees suggests that the modes of action identified here may also occur in bees^1,21,23,33,53–56^. Previous work has shown that sub-lethal effects observed in the lab translate to the field, for example neonicotinoid reduced foraging motivation is observed in bumblebees in the lab as well as in free flying colonies in the field^20,66^. Reduced behavioural rhythmicity, shown in honeybees^23,33^, bumblebees^21^ and here in fruit flies, is likely to reduce the amount of activity carried out in the daytime, reducing pollination and foraging opportunities^67^. The reduction in total sleep and fragmentation of sleep will reduce the quantity of deep sleep achieved, as deep sleep occurs later into the sleep episode^68^. It is known that deep sleep is particularly important for memory consolidation^32^, therefore this is likely to further compound the effects of neonicotinoids on memory, which will again impact foraging efficiency.

In summary, nAChR subunits Dα1 and Dβ2 expression in the mushroom body appears to mediate the effect of field-relevant concentrations of nitro-containing imidacloprid, clothianidin and thiamethoxam on memory, while expression of these subunits in the clock mediates the effect of neonicotinoids on circadian rhythmicity and sleep. The more recently banned cyano-containing neonicotinoid thiacloprid was less toxic, only disrupting sleep, which seems to be the most sensitive behavioural metric of the sub-lethal effects of neonicotinoids. In addition, all four of the neonicotinoids tested decreased both viability and shortened lifespan, therefore supporting the current EU ban. This work illustrates the utility of neonicotinoids as a pharmacological tool for exploration of nAChR function, as well as the use of *Drosophila* in revealing the mechanism of action of neonicotinoids and elucidating the sub-lethal and lethal effects of these insecticides, highlighting their potential impact on insects in the field.

## Materials and methods

### Fly husbandry and genotypes

The following fly stocks were used: wild-type strains *iso31* (Gift from Dr Ralph Stanewsky, University of Münster, Germany) for climbing, circadian and sleep assays and *CSw-* (gift from Dr Scott Waddell, University of Oxford, UK) for all other experiments, *Pdf-Gal4* (Bloomington *Drosophila* stock center number (BDSC): 6900), *elav-Gal4* (BDSC: 8760), *tim*^28^*-Gal4* (BDSC: 80976, gift were from Dr Ralf Stanewsky, University of Münster, Germany), *OK107-Gal4* (BDSC: 854), *uas-mcd8::GFP* (BDSC: 5137), *uas-nAChR-Dα1-RNAi* (BDSC: 28688), and *UAS-nAChR-Dβ2-RNAi* (BDSC: 28038). For all experiments, flies were collected shortly after eclosion and used within 5 days. For climbing, circadian, sleep, longevity, immunohistochemistry and offspring survival assays females were used, for learning and memory mixed sex groups were used.

Flies were bred, maintained and tested on standard polenta-based food mixture at 25 °C, 55–65% humidity under 12 h light:12 h dark (LD) conditions. Food was made up in 5 L quantities and contained: 400 g polenta, 35 g granulated agar, 90 g active dried yeast, 50 g soya flour, 400 mL malt extract and 200 mL molasses, with 40 mL of propionic acid (Sigma-Aldrich, #94425) and 100 mL of nipagin (Sigma-Aldrich, #H5501) added once cool. Neonicotinoids were added to food before it set from a frozen and aliquoted stock solution of 100,000 μg/L ddH_2_O. The neonicotinoids were analytical standard (PESTANAL Sigma-Aldrich): imidacloprid, clothianidin, thiamethoxam, and thiacloprid.

### Aversive olfactory conditioning

1 h memory was tested using aversive olfactory conditioning^57^. Groups of 30–50 one-five days old mixed sex flies were reared on control or neonicotinoid containing food. Flies were exposed consecutively to one of two odours, either 4-methylcyclohexanol (Sigma) or 3-octanol (Sigma) diluted 1:500 and 1:250 respectively, paired with 1.25 s pulses of 70 V electric shock, with 3.75 s pauses between shocks. Flies were then returned to food vials for 1 h before memory was tested. For testing, flies were loaded into the choice point of the T-maze and, after a 90 s acclimatisation period, were given the choice of two tubes, one containing each of the test odours. Flies were given 2 min and then the proportion in each arm was counted. A separate group of flies were then trained and tested with the reciprocal odour. The performance index score represents the proportion of flies who correctly avoided the arm containing the odour which had been delivered with shock during training, as shown below.

PI = (number of correct flies − number of incorrect flies)/total number of flies.

The PI for flies shocked with each odour separately were averaged to give an *n* = 1.

### Circadian rhythms

Behavioural rhythmicity data was collected using *Drosophila* Activity Monitors (DAM2, TriKinetics Inc)^69^. Virgin females were placed in individual tubes in the DAM, with control or neonicotinoid containing food, 32 flies per treatment, for 5 days in LD followed by 5 days constant darkness (DD). Flies who died before the end of day ten were not included in analysis. DAM tubes were intersected with an infrared beam, with each beam cross counted as an activity bout. To quantify circadian rhythmicity, the data was summed into 30 min bins. From this an actogram was created and rhythmicity statistic and period length were calculated for each individual fly for the DD portion using Flytoolbox^70^ in MATLAB (MATLAB and Statistics Toolbox Release 2012b, The MathWorks). The proportion of flies in each treatment group whose Rhythmicity Statistic was below 1.5, generally considered to denote arrhythmicity^70^, was calculated. This was then displayed in a pie chart for each group, after being normalised by removing the proportion of controls who were arrhythmic for each run. The rhythmicity of each fly during the LD section of the run was also calculated.

### Sleep

For analysis of sleep behaviour, flies were loaded into the DAM as described above. Sleep measures were extracted from activity data from 5 days of LD, summed into 1 min and 30 min bins. Sleep was defined as bouts of inactivity lasting more than 5 min, as is convention^70,71^. The mean total sleep, mean sleep episode length, mean number of sleep episodes per day and night and mean sleep latency were calculated for each individual using the Sleep and Circadian Analysis MATLAB Program (SCAMP) in MATLAB^72^. For each treatment, a mean sleep profile was also plotted showing mean sleep quantity per 30 min bin over the 24 h period.

### Imaging the arborisation and PDF cycling of s-LNv dorsal terminals

Immunohistochemistry was adapted from the method described in Fernández et al.^45^. Virgin females were collected and placed in vials. After 5 days LD, on control or neonicotinoid food, flies were anesthetised using CO_2_ at either 2 h after lights on (ZT2) or 2 h after lights off (ZT14) and decapitated. Heads were fixed in phosphate-buffered solution (PBS) with 4% paraformaldehyde (Thermo Fisher Scientific) containing 0.008% Triton X-100 (Sigma-Aldrich) for 45 min at room temperature. Heads were washed quickly twice in PBS with 0.5% Triton X-100 (PBT 0.5%), followed by three 20 min washes in PBT 0.5% and dissection in PBT 0.1%. Brains were blocked in 5% Normal Goat Serum (NGS, Thermo Fisher) for 1 h, then incubated for 36 h at 4 °C with mouse monoclonal anti-PDF (Developmental Studies Hybridoma Bank, #PDF-C7) and rabbit polyclonal anti-GFP (Life Technologies # A11122) in NGS at concentrations of 1:200 and 1:1000 respectively.

Brains were washed again as before and then incubated for 3 h at room temperature followed by 24 h at 4 °C with Alexa Fluor Plus 488 Goat anti-mouse (Life Technologies # A32723) and Alexa Fluor Plus 555 goat anti-rabbit (Life Technologies # A32732) in NGS at concentrations of 1:1000 and 1:100 respectively. Brains were washed once more and then mounted onto glass slides using spacers (SecureSeal, Grace Bio-Labs #654002), covered with VectaShield hard set medium (Vector Laboratories) and secured with CoverGrip (Biotium #23005).

Imaging was carried out on a Leica SPE confocal laser scanning microscope with the green channel imaged at 480–551 nm and the red at 571–650 nm. Z stacks were captured of the s-LNv dorsal terminals using a 64 × oil immersion objective, with a step size of 2 µm. Maximal projection stacks were created and analysed using FIJI (ImageJ)^73^. The arborisation of each s-LNv terminal was calculated using an adaptation of the Scholl analysis^45^. Six concentric rings 10 μm apart were drawn, centred at the first branching point and the number of branches touching each ring was summed. Both hemispheres were measured. Mean scores for ZT2 and ZT14 in each group were compared using Pearson’s *t*-test using SPSS Statistics 24 (IBM Corporation).

For PDF staining intensity, the image was cut at the first branching point to create an image containing only the terminals and not the cell axon and the image was analysed in FIJI (ImageJ). The image was transformed into 8-bit and the threshold adjusted to create a black and white image. The de-speckle filter was used to reduce noise and watershed segmentation carried out to separate the different PDF compartments. This image was then used as a template for calculating the PDF staining in the original maximal projection image, allowing the PDF staining intensity to be calculated for each of these compartments and the mean taken. The mean for both hemispheres of the brain was calculated and reported, and the means for ZT2 and ZT14 were compared as for axonal branching.

### Statistical analysis

Normality of the data was checked using a Shapiro–Wilk test. Data were also checked for homogeneity of variance using Levene’s test for equality of variances. Unless otherwise stated, means were then compared using a one-way ANOVA with post hoc pairwise comparisons being carried out using Tukey’s multiple comparisons test. Where data were not normally distributed, a Kruskal–Wallis one-way ANOVA was carried out with Dunnett’s multiple comparison. Statistical analysis was done in SPSS Statistics 24 (IBM Corporation). Graphs were created in GraphPad (Prism version 8.0.0 for Windows, GraphPad Software).

The sleep data failed the assumptions of normal distribution and homogeneity of differences. Thus, permutation tests were conducted in R 3.4.1. As the resulting *p* values closely matched those produced by analysing the same data using a one-way ANOVA as above, and because ANOVA is relatively robust to deviations from normality when sample sizes are large, the results of the one-way ANOVA were displayed.

## Supplementary Information

Supplementary Information.

## Data Availability

The datasets generated during and/or analysed during the current study are available in the Mendeley Data repository, under ‘Neonicotinoids disrupt memory, circadian behaviour and sleep, Tasman et al.’, https://doi.org/10.17632/d28dk6znm6.1
