# Supplementary material for: Neonicotinoids disrupt memory, circadian behaviour and sleep
Source: Sci Rep. 2021 Jan 21;11:2061. doi: 10.1038/s41598-021-81548-2 (PMC7820356; doi:10.1038/s41598-021-81548-2)
Supplement: Supplementary file 1 — Supplementary Information. [file 41598_2021_81548_MOESM1_ESM.docx]

**Neonicotinoids disrupt memory, circadian behaviour and sleep**

**Kiah Tasman^1^, Sergio Hidalgo^1^, Bangfu Zhu^1^, Sean A. Rands^2^, James J.L. Hodge^1,^***

**Affiliations: ^1^**School of Physiology, Pharmacology and Neuroscience, University of Bristol
Biomedical Sciences building, University Walk, Bristol, BS8 1TD, UK

**^2^**School of Biological Sciences, University of Bristol, Life Sciences Building, Tyndall Avenue, Bristol, BS8 1TQ, UK

**Corresponding author:** [***james.hodge@bristol.ac.uk**](mailto:*james.hodge@bristol.ac.uk)

**Supplementary information:**

**Methodology:**

**Longevity**

Ten once mated, one day old females were placed in a vial containing control or neonicotinoid containing food and transferred into a fresh vial every 2 days with the number of dead flies noted. This was continued until all flies were dead [1] with ten repeats being performed per treatment group. A survival curve was created and analysed using GraphPad (GraphPad Prism version 6.05 for Windows, GraphPad Software) and mean lifespan calculated. The difference of the treatment survival curve from the control survival curve was analysed using a log-rank (Mantel-Cox) test.

**Offspring viability**

Flies were reared on control or neonicotinoid containing food. Ten groups of ten once mated females were collected, and then each female was placed in a vial of control fly food and allowed to lay eggs over a 24hour period. The number of eggs in each vial was quantified and then compared to the number of adult flies which successfully eclosed from the vial ~15 days later, giving a % offspring survival for each group [2].

**Locomotor assay**

Climbing ability was used as a measure of locomotion of adult flies and was determined by the negative geotaxis assay, whereby flies were tapped to the bottom of a tube, causing them to move away from gravity (negative geotaxis). Twenty-five groups of ten females were placed in vials of control or neonicotinoid containing food for 5 days. They were then placed into empty vials. After 5 minutes of acclimatisation, flies were knocked to the bottom of the vial and given 10 seconds to climb [3]. The performance index represents the proportion of flies who successfully climbed ≥7 cm in 10 seconds.

**Sensory Controls**

Sensory controls were carried out to check the capacity of treatment groups to sense olfactory and shock cues. For olfactory acuity, groups of 1-5 day old 30-50 mixed sex flies, reared on control or neonicotinoid food, were loaded into the T-maze and provided with a choice between an odour (1:500 4-methylcyclohexanol or 1:250 3-octanol) and fresh air. For shock reactivity, similar groups of flies were given a choice between two shock tubes, one of which was delivering 1.5 second pulses of 70 V electric shock, with 3.5 second pauses between shocks. In both cases, flies with normal sensory capacity should avoid the stimuli. The proportion of each group who avoided the odour or shock was reported.

**qPCR**

Relative measure of Drosophila nAchR α1(Dα1) and nAchR β2(Dβ2) subunit expression levels was assessed by two-step qPCR. 2–5 days old ﬂies were anesthetized with CO2 and decapitated, obtaining three biological replicates with 30 heads each. Total RNA was extracted from head lysates by organic phenol/chloroform method using TRIzol reagent (Invitrogen). RNA quantiﬁcation was carried out in Nanodrop spectrophotometer (Thermo Scientiﬁc) and samples were treated with TURBO DNA-free kit (Invitrogen) in order to remove genomic DNA contamination. Reverse transcription was carried out using RevertAid First Strand cDNA Synthesis Kit (Thermo Scientiﬁc) following manufacturer’s instructions, with 1000 ng of RNA as template and Oligo (dT) as primer to amplify total mRNA. cDNA samples were stored at −20◦C or used immediately for qPCR reactions. Quantitative PCR reactions were carried out in QuantStudio 3 Real-Time PCR system (Applied Biosystems) using PowerUp SYBR Green Master Mix (Applied Biosystems).

Quantiﬁcation for each genotype and each gene was carried out using the 2(−ΔΔCt) method to normalize with the Drosophila actin (42A) endogenous gene and data was expressed as a percentage of change (Mean+SEM). Relative levels of mRNA were analyzed by one-way ANOVA analysis with Tukey’s multiple comparisons test performed using GraphPad Prism 8.0 software.

The following primers were used:

Actin (F): 5’- GTGTGCAGCGGATAACTAGAA-3’,

Actin (R): 5’- ATCCGTTGTCGACCACTAAAG-3’.

nAchR α1(F):5’- CTAAGATGGGTAGCGTGCTATT-3’,

nAchR α1(R): 5’- CGGATGAGGCGATTGTAGTT-3’.

nAchR β2(F): 5’- CGAATTCCACGGCTCCAATA-3’,

nAchR β2(F): 5’- TGTTCACAACGGGCCTAATC-3’

**
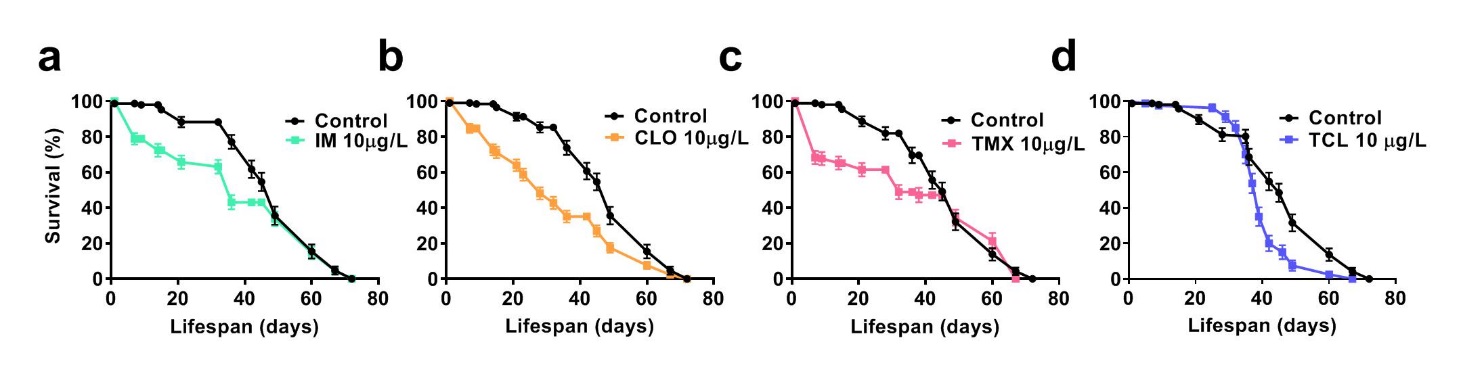

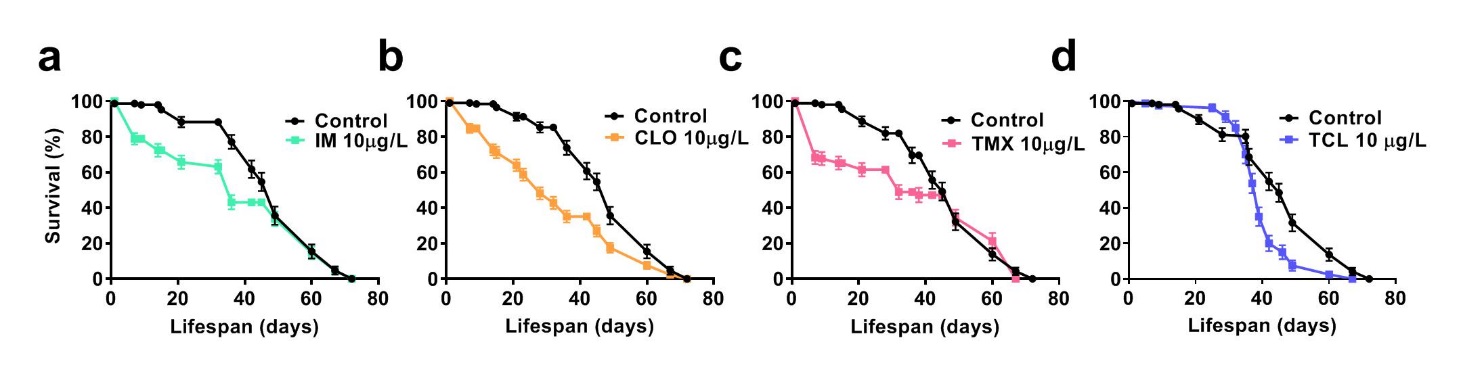

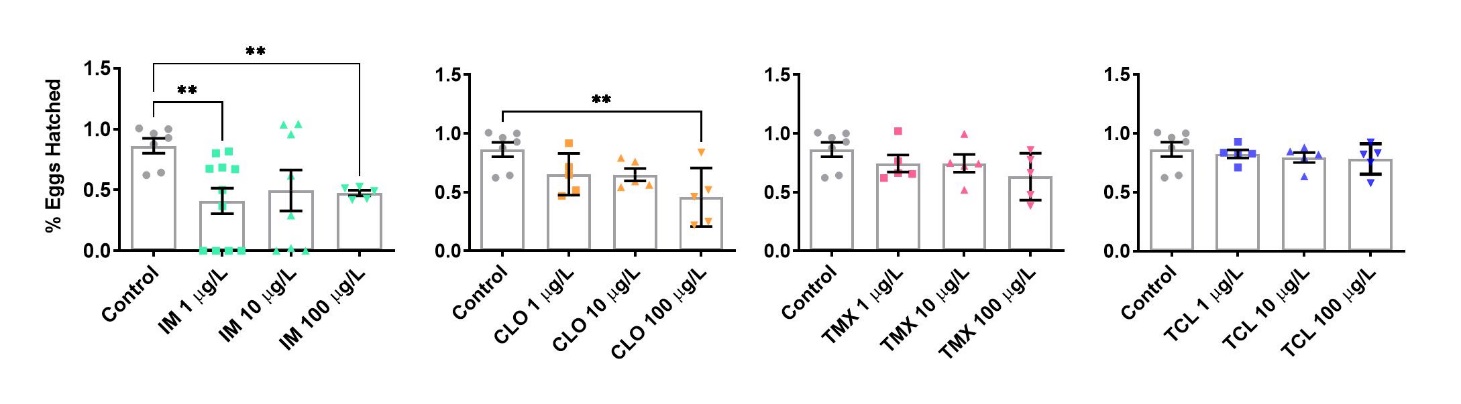

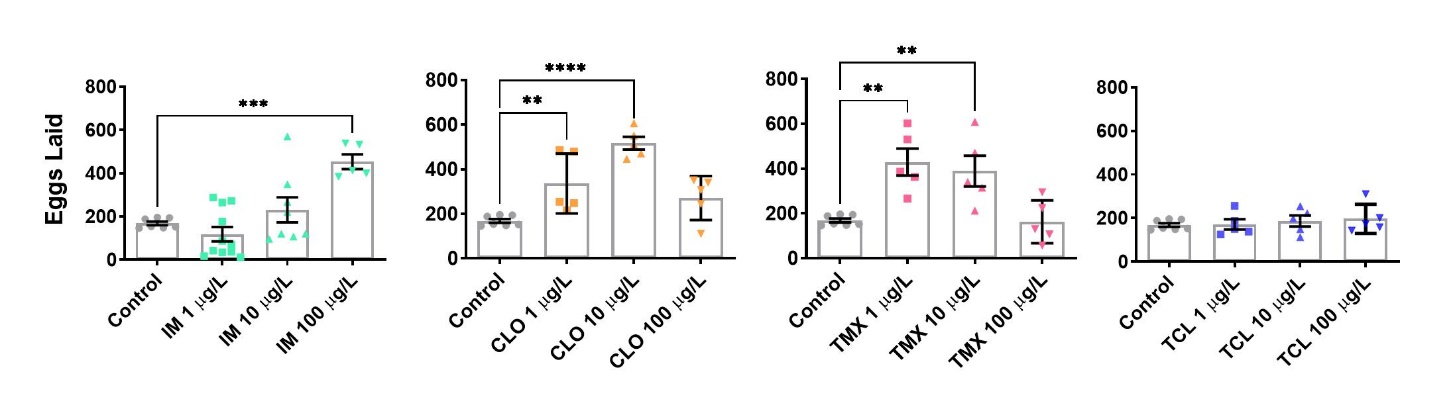

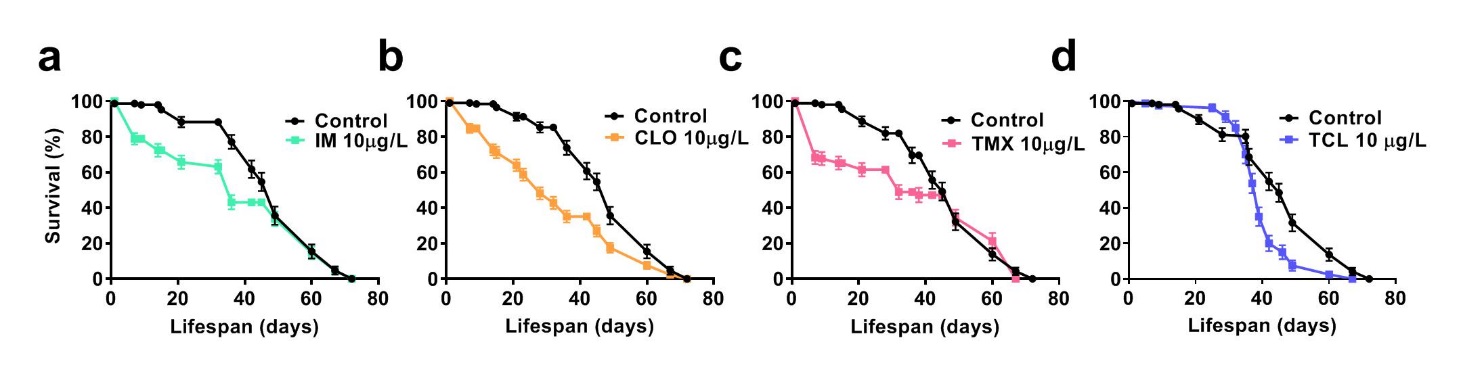
**

**Supplementary Figure S3| Field-relevant concentrations of neonicotinoids reduce the % eggs hatched.** The viability of eggs of flies exposed to 1, 10 or 100 μg/L of **a**, IM (*W*_3,14_ =10.91, *p* ≤ 0.001), **b,** CLO (*F*_3,18_ = 4.99, *p* =0.011), **c,** TMX (*F*_3,18_ = 1.76, *p* = 0.191) and **d**, TCL (*F*_3,18_ = 0.50, *p* = 0.687), *n*=5-11 groups of 10 once mated female flies for each treatment. Viability was measured by counting the number of eggs laid by 10 once mated female in 24 h period and then counting the % of those eggs that completed development, eclosing adults ~15-18 days later.

**Supplementary Figure S2| Field-relevant concentrations of neonicotinoids increase egg laying.** The egg laying of flies exposed to 1, 10 or 100 μg/L of **a**, IM (*F*_3,27_ = 10.82, *p* ≤ 0.001), **b,** CLO (*F*_3,18_ = 16.87, *p* <0.001), **c,** TMX (*F*_3,18_ = 9.50, *p* < 0.001) and **d**, TCL (*F*_3,18_ =0.40, *p* = 0.649), *n*=5-11 groups of 10 once mated female flies for each treatment.

**Supplementary Figure S1| Field relevant concentrations of neonicotinoids reduced longevity.** Compared to the median lifespan for control flies (49 days) flies exposed to 10 μg/L of **a,** IM (36 days,( *χ^2^*_1_=8.3_,_ *p*=0.004), **b,** CLO (28 days, (*χ^2^*_1_=41.0, *p* <0.001), **c,** TMX (36 days, (*χ^2^*_1_=5.8, *p*=0.016) and **d,** TCL (39 days, (*χ^2^*_1_=18.5, *p* <0.001) had shorter lives. *n*=100 flies for each group.


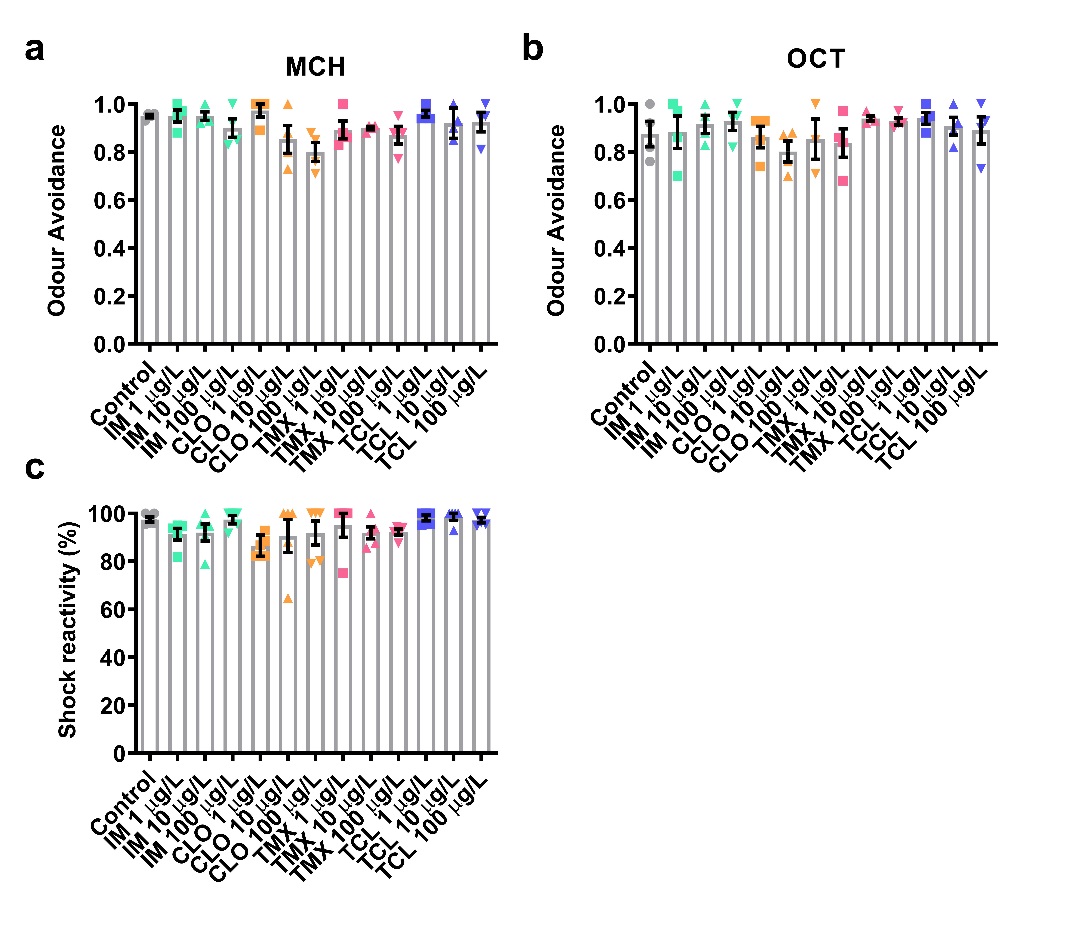

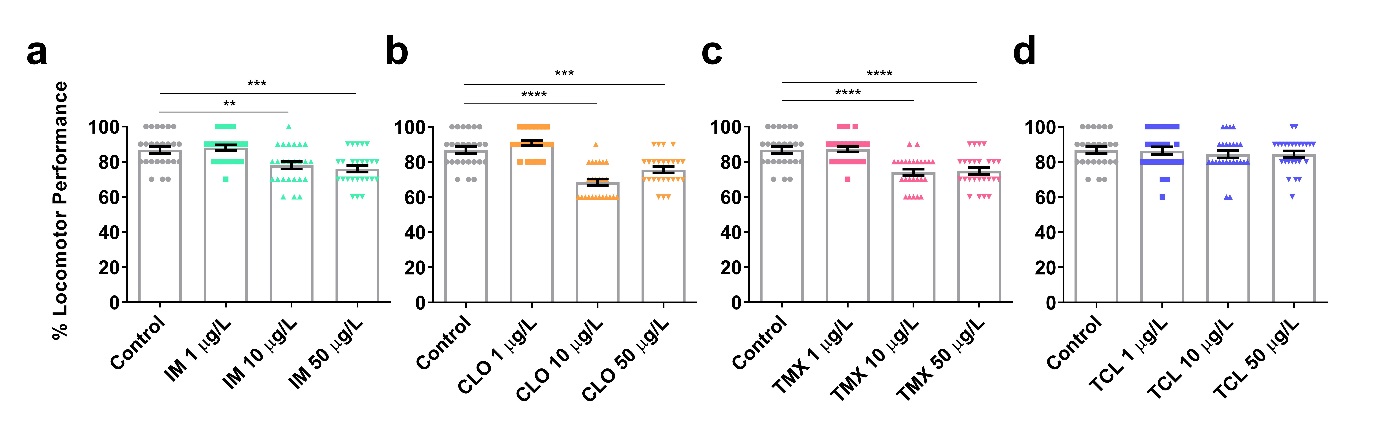


**Supplementary Figure S5| Field relevant concentrations of neonicotinoids do not disrupt olfaction or shock reactivity.** Sensory controls for olfactory-shock conditioning memory assays (Fig. 1) show 1, 10 and 100 μg/L of IM, CLO, TMX and TCL did not affect **a,** odour avoidance of 4-methylcyclohexanol (MCH) (*χ^2^*_12_ = 19.5, *p* = 0.076), or **b,** 3-octanol (OCT) (*χ^2^*_12_ = 10.0, *p* = 0.674) and **c,** shock reactivity (*χ^2^*_12_ = 22.6, *p* = 0.031). Each data point represents a group of ~50 flies, tested together, *n*=4 for each group.

**Supplementary Figure S4| Field relevant concentrations of neonicotinoids reduce locomotor performance.** Locomotor performance was measured using the negative geotaxis climbing assay, flies exposed to 10 or 50 μg/L of the banned neonicotinoids: **a,** IM (*F*_3,96_ = 9.9, *p* <0.001), **b,** CLO (*F*_3,96_ = 32.0, *p* <0.001), **c,** TMX (*F*_3,96_ = 15.8, *p* <0.001) significantly reduced climbing performance, while the non-banned neonicotinoid **d,** TCL (*F*_3,96_ = 0.4, *p* = 0.762) did not affect locomotion. *n*=25 groups of 10 flies for each treatment group.

**
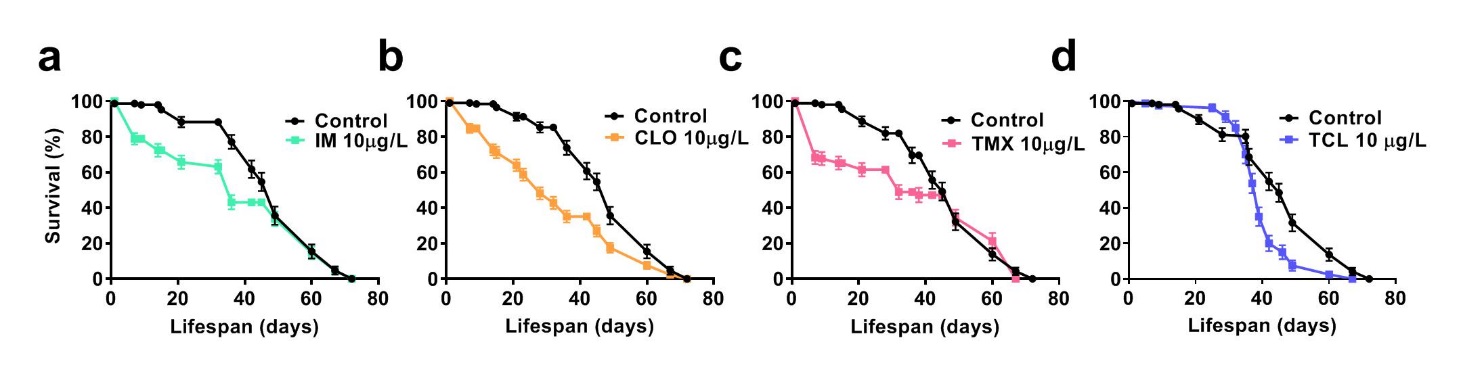
**
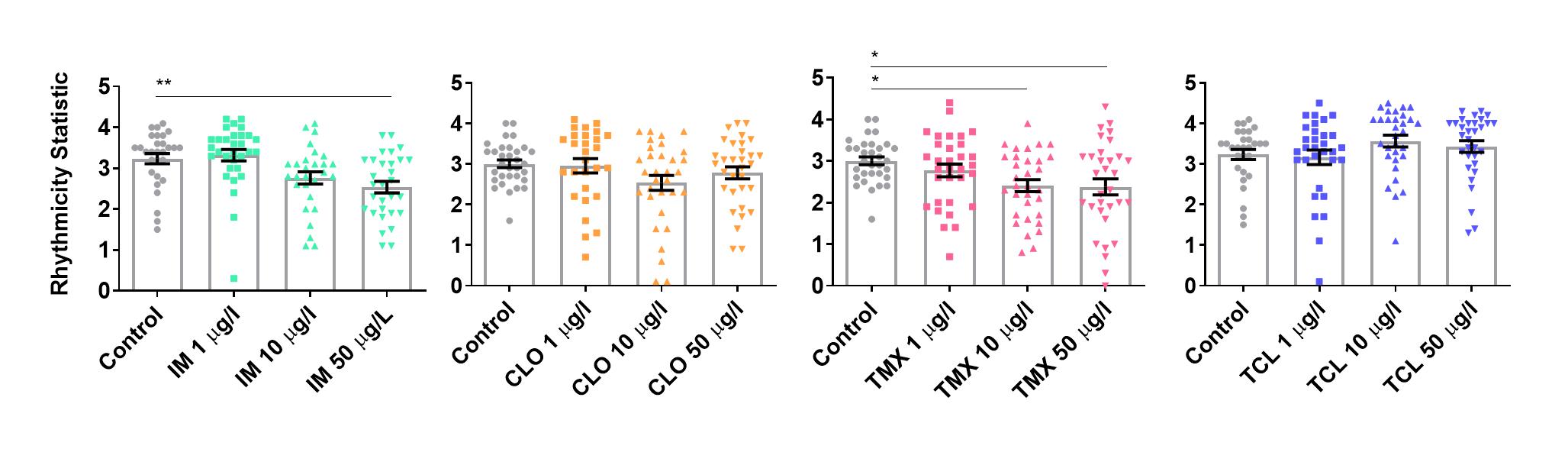
**
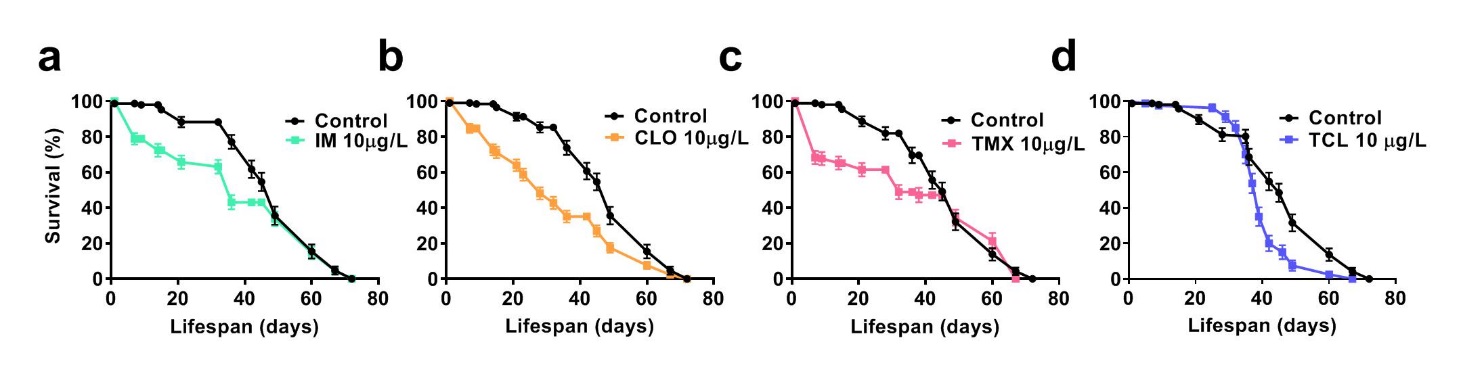
**
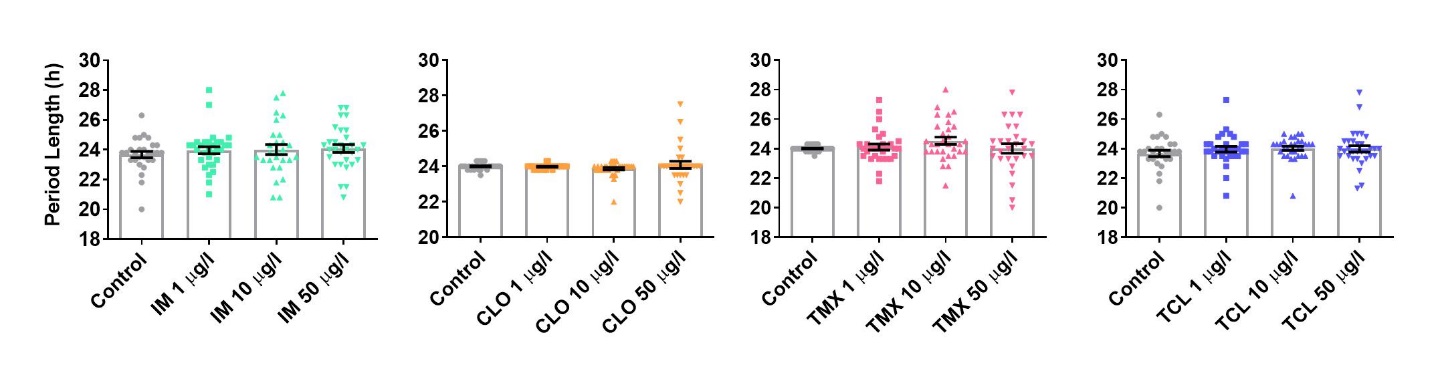


**Supplementary Figure S7| Field relevant concentrations of neonicotinoids can affect rhythmicity in 12:12 light:dark conditions.** The rhythmicity statistic for flies exposed to 1, 10 or 50 μg/L of **a,** IM (*F*_3,114_=7.27, p < 0.001), **b,** CLO, (*F*_3,120_=1.93, p=0.128), **c,** TMX, (*F*_3,117_=4.03, p=0.009) or **d,** TCL, (*F*_3,118_=1.51, p=0.217). Each datapoint represents a single fly, n=28-32 flies per treatment.

**Supplementary Figure S6| Field relevant concentrations of neonicotinoids do not affect period length under constant conditions.** The length (h) of the free running period in constant darkness, for flies exposed to 1, 10 or 50 μg/L of **a,** IM (*F*_3,112_=0.44, p=0.722), **b,** CLO, (*F*_3,116_=0.73, p=0.536), **c,** TMX, (*F*_3,117_=1.36, p=0.258) or **d,** TCL, (*F*_3,118_=0.65, p=0.584). Each datapoint represents a single fly, n=28-32 flies per treatment.


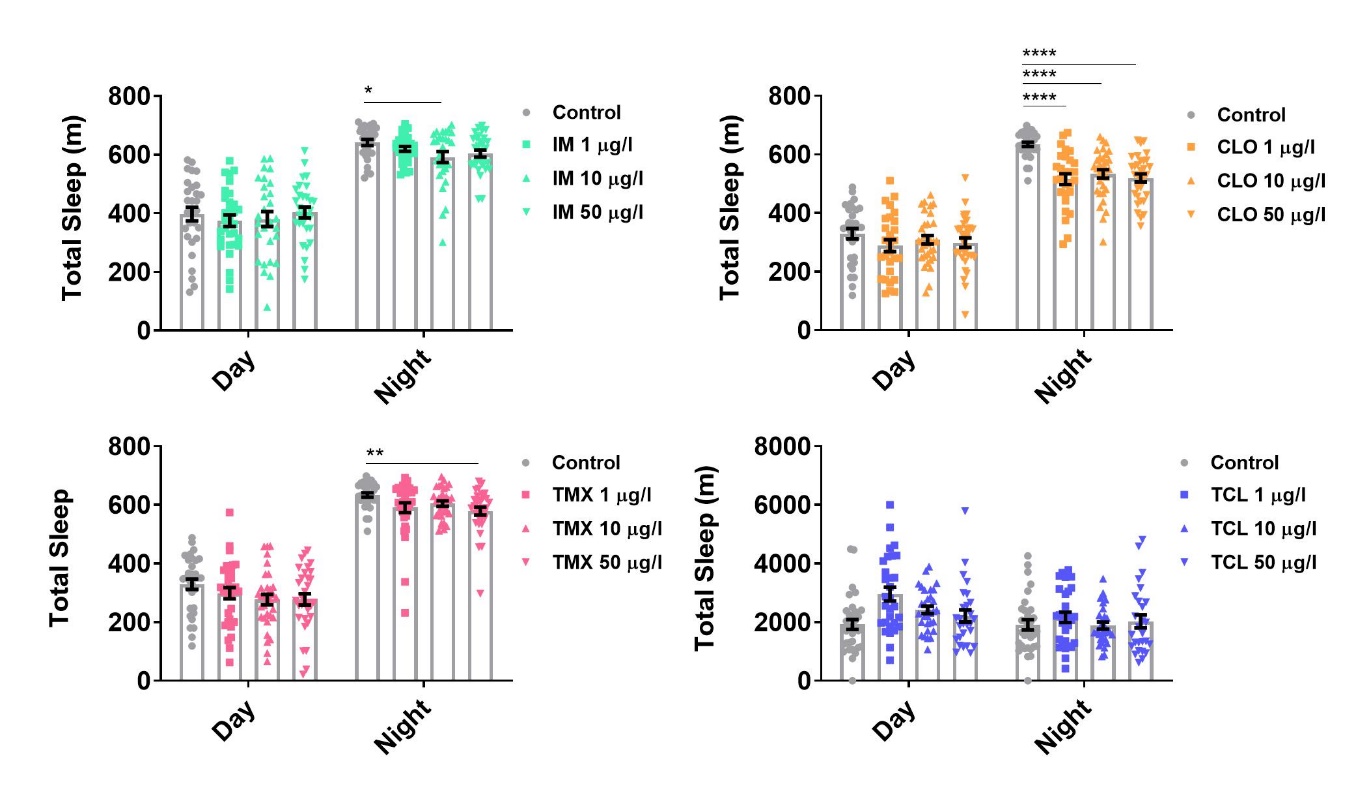
**
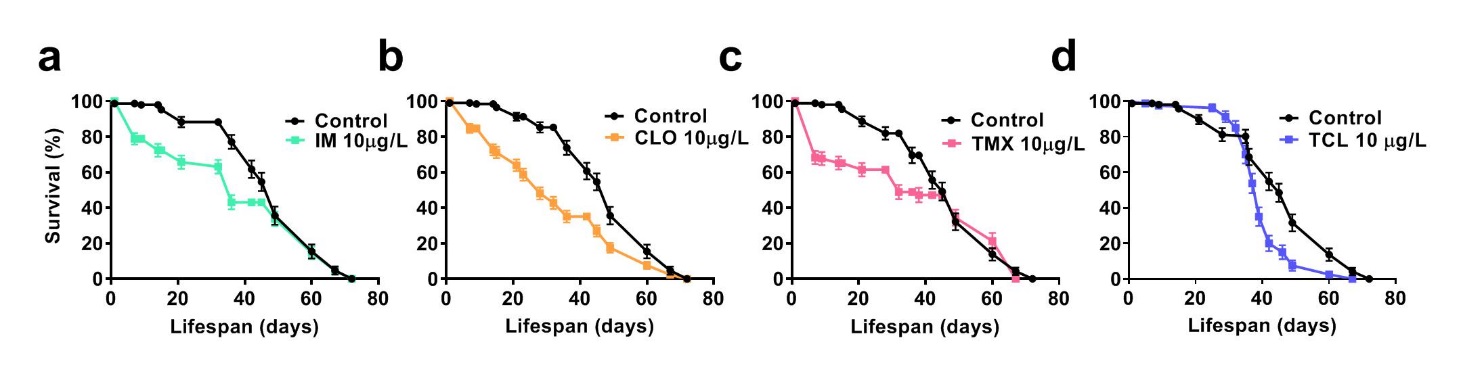
**
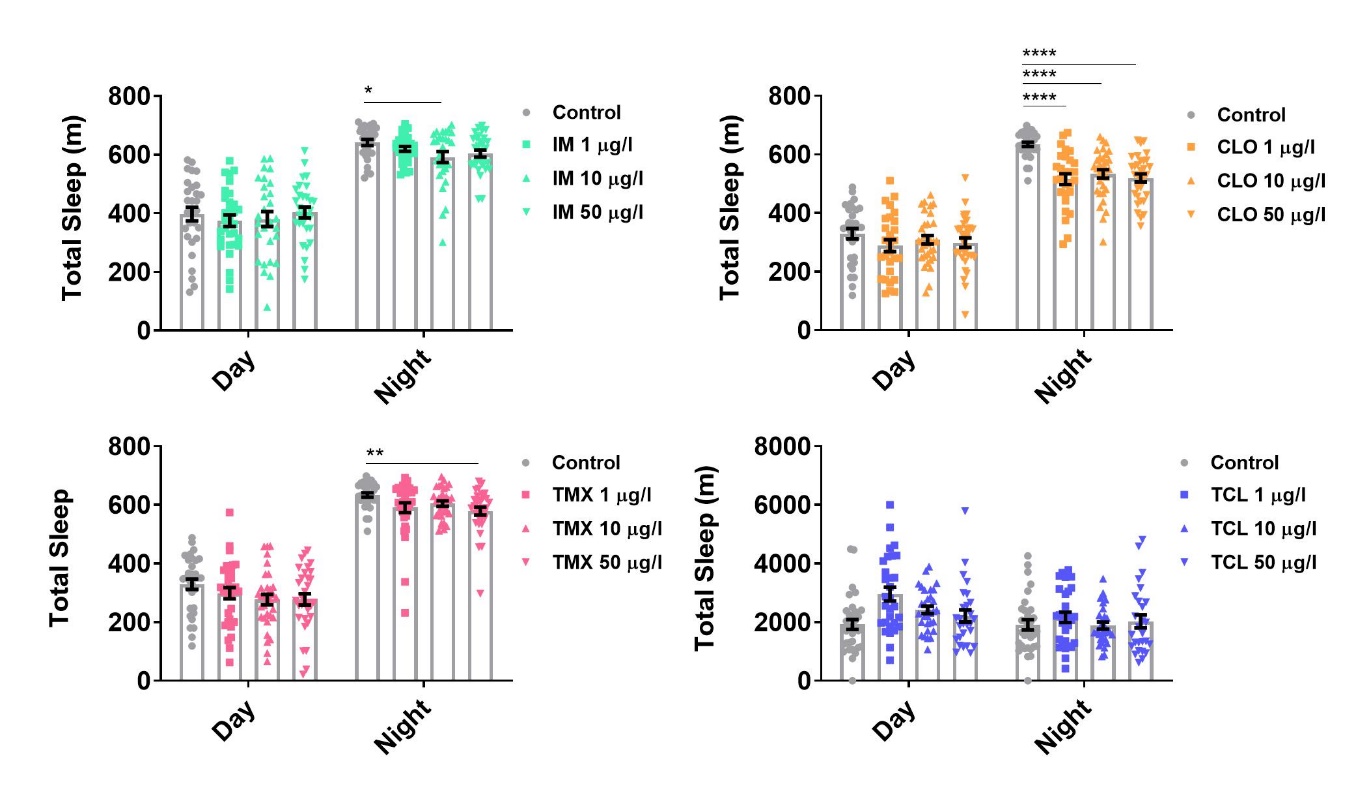
**
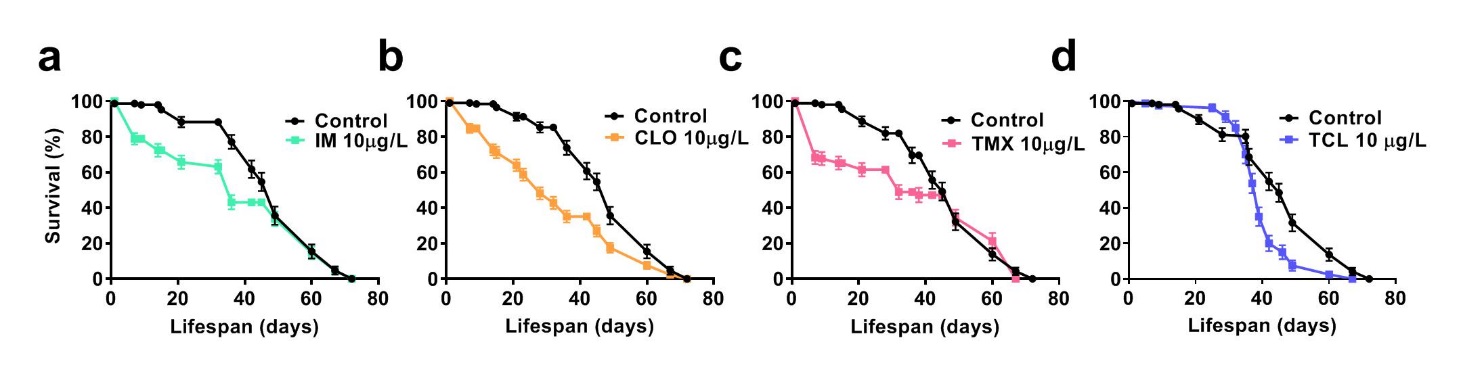

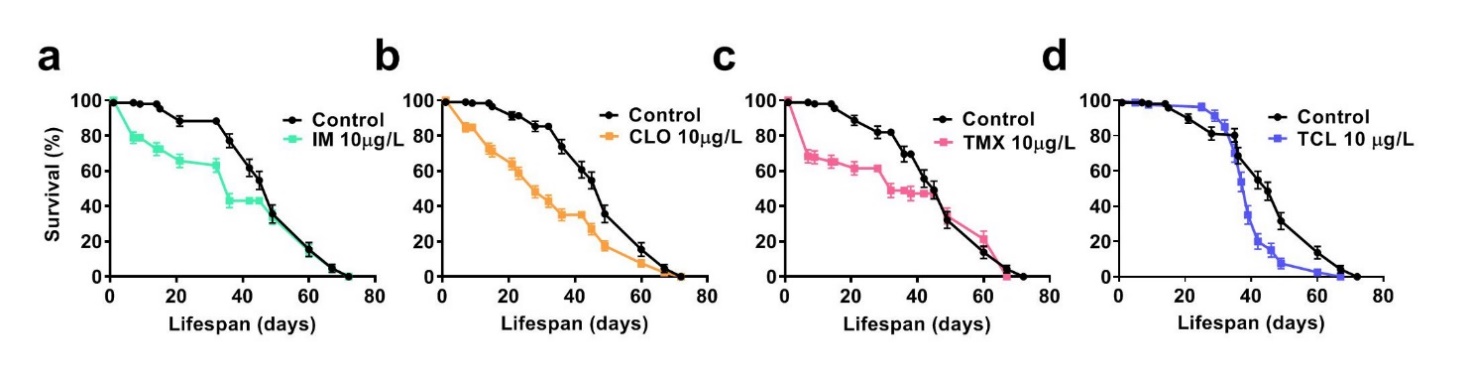

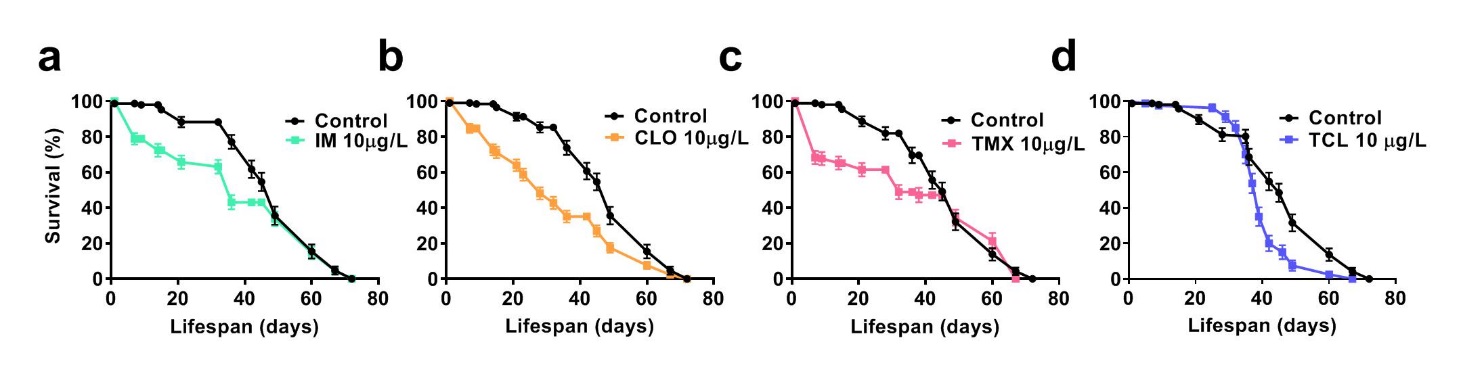
**
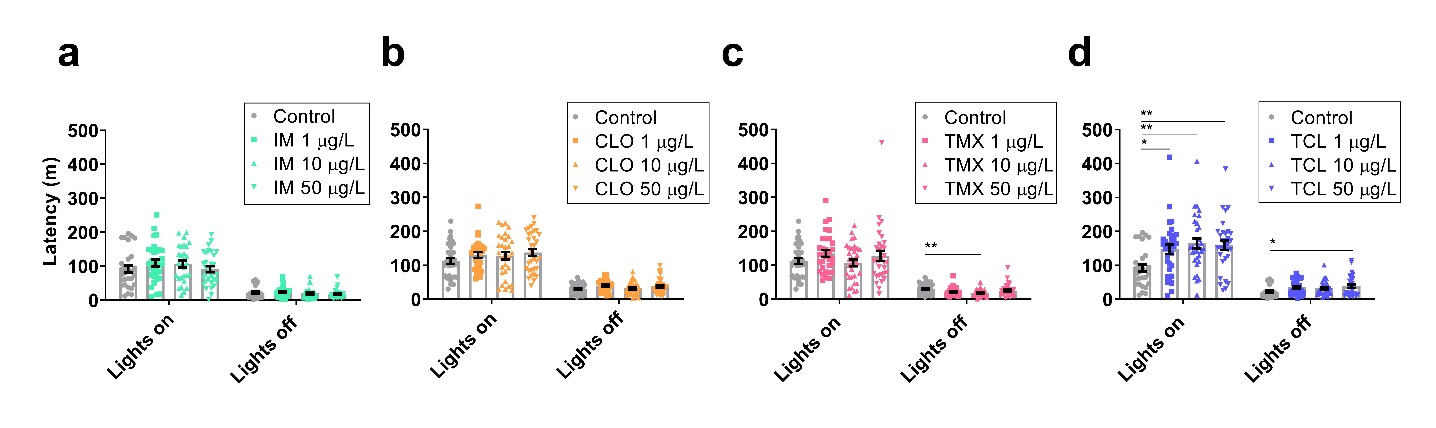

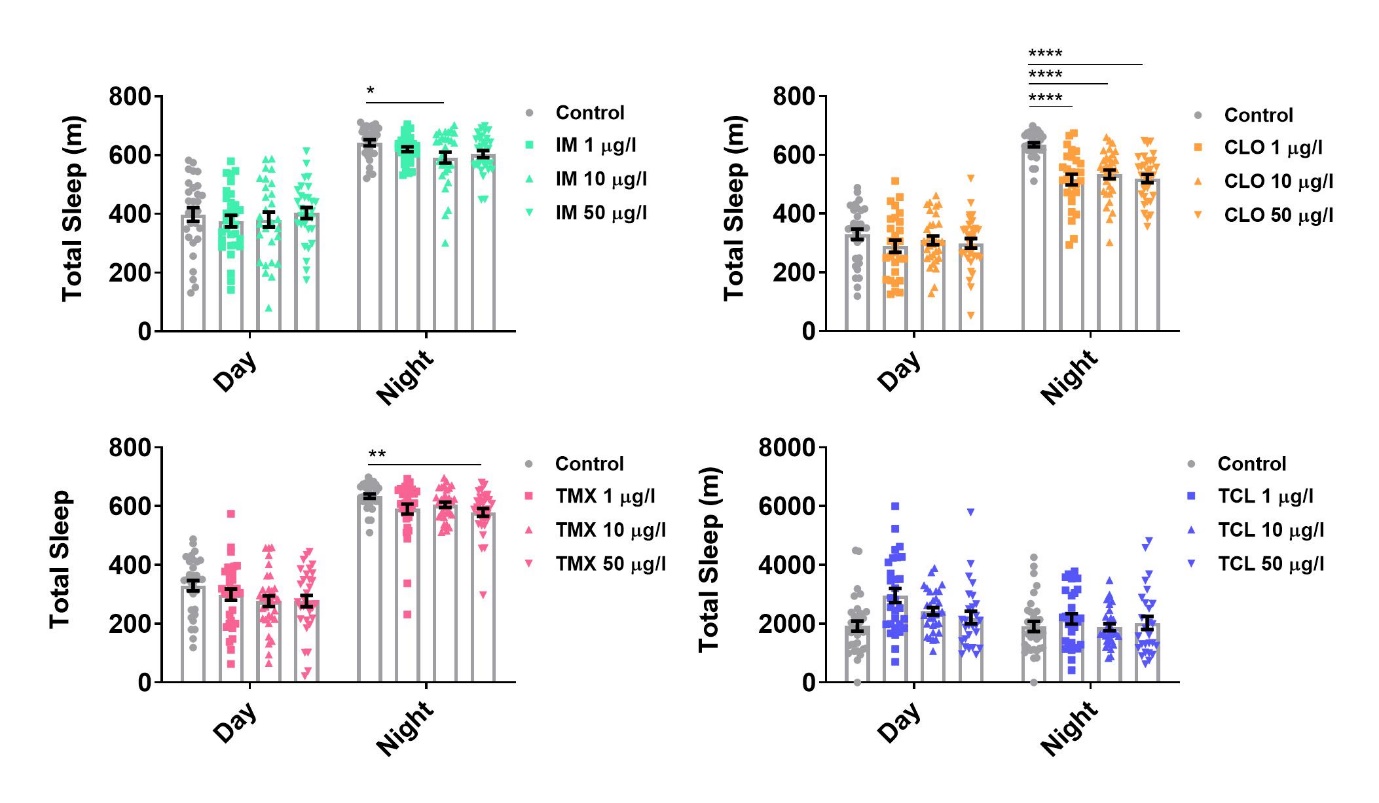


**Supplementary Figure S9| Field relevant concentrations of thiacloprid increase daytime sleep latency.** The mean latency in minutes (m) before sleep was initiated after lights on or lights off, for flies exposed to 1, 10 or 50 μg/L of **a,** IM, day (*F*_3,114_=0.9, p=0.441) and night (*F*_3,114_=0.9, p=0.468), **b,** CLO, day (*F*_3,120_=1.1, p=0.333) and night (*F*_3,120_=2.0, p=0.124), **c,** TMX, day (*F*_3,124_=1.3, p=0.264) and night (*F*_3,124_=4.3, p=0.007) or **d,** TCL, day (*F*_3,120_=5.6, p=0.001) and night (*F*_3,120_=2.7, p=0.50). Each datapoint represents a single fly, n=28-32 flies per treatment.

**Supplementary Figure S8| Field relevant concentrations of neonicotinoids can reduce total sleep.** The total quantity of sleep achieved (m) for flies exposed to **a,**imidacloprid-IM, day (*F*_3,114_)=0.4, p=0.772) and night (*F*_3,114_ = 2.9, p=0.040), **b**, clothianidin-CLO, day (*F*_3,120_=1.0, p=0.378) and night (*F*_3,120_=16.6, p≤0.001), **c**, thiamethoxam-TMX, day (*F*_3,124_=1.8, p=0.154) and night (*F*_3,124_=3.7, p=0.013) or **d**, thiacloprid-TCL, day (*F*_3,120_=4.5, p=0.005) and night (*F*_3,120_=1.8, p=0.157). Each datapoint represents a single fly, n=28-32 flies per treatment.


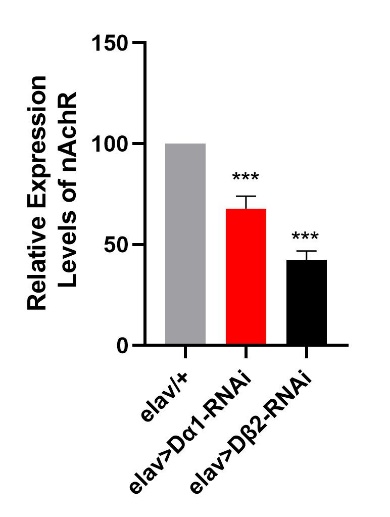


**Supplementary Table S1| Field relevant concentrations of neonicotinoids increase the proportion of the population (%) exhibiting arrhythmicity compared to controls**

**Supplementary Figure S10| RNAi mediated knock-down of Dα1 or Dβ2 results in significantly lower expression of Dα1 or Dβ2 mRNA respectively.** The mRNA expression levels of Drosophila nAchR subunits α1(Dα1) and β2(Dβ2) upon RNAi knockdown. Compared to pan-neuronal expression control (elav/+), expressing an RNAi against Dα1 or Dβ2 reduced its expression significantly by 33 % and 58 %, respectively. (***p < 0.001.)

(elav/+: Elav-Gal4/+; elav>Dα1-RNAi: elav-Gal4>uas-nAChR-Dα1-RNAi; elav> Dβ2-RNAi: elav-Gal4>uas-nAChR-Dβ2-RNAi.)

|  | IM | CLO | TMX | TCL |
| --- | --- | --- | --- | --- |
| 1 µg/L | 8% | 11% | 10% | 1% |
| 10 µg/L | 19% | 19% | 23% | 4% |
| 50 µg/L | 27% | 36% | 65% | 0% |

**References:**

1 Lowe, S. A., Usowicz, M. M. & Hodge, J. J. L. Neuronal overexpression of Alzheimer's disease and Down's syndrome associated DYRK1A/minibrain gene alters motor decline, neurodegeneration and synaptic plasticity in *Drosophila*. *Neurobiology of disease* **125**, 107-114, doi:10.1016/j.nbd.2019.01.017 (2019).

2 Grönke, S., Clarke, D.-F., Broughton, S., Andrews, T. D. & Partridge, L. Molecular evolution and functional characterization of *Drosophila* insulin-like peptides. *PLOS Genetics* **6**, e1000857, doi:10.1371/journal.pgen.1000857 (2010).

3 Nichols, C. D., Becnel, J. & Pandey, U. B. Methods to assay *Drosophila* behavior. *Journal of Visualized Experiments : JoVE*, **61** 3795, doi:10.3791/3795 (2012).
